# Design, Synthesis, and In Silico Insights of new 4‐Piperazinylquinolines as Antiproliferative Agents against NCI Renal Cancer Cell Lines

**DOI:** 10.1002/open.202400518

**Published:** 2025-02-05

**Authors:** Gabriele La Monica, Alessia Bono, Federica Alamia, Annamaria Martorana, Antonino Lauria

**Affiliations:** ^1^ Department of Biological Chemical and Pharmaceutical Sciences and Technologies (STEBICEF) University of Palermo Viale delle Scienze, Ed. 17 I-90128 Palermo Italy

**Keywords:** antiproliferative activity, molecular hybridization, piperazine, quinoline, structure-based studies

## Abstract

In the search for new anticancer compounds, quinoline and piperazine moieties represent the most promising pharmacophoric fragments for the development of more effective drugs. A particularly interesting approach in medicinal chemistry is molecular hybridization, where different known components are integrated into a single chemical entity, resulting in hybrid molecules with enhanced biological activity. In this study, we have developed a new series of 4‐(4‐benzoylpiperazin‐1‐yl)‐6‐nitroquinoline‐3‐carbonitrile compounds (**8 a**–**l**), with potential anticancer effect, by combining the quinoline, the piperazinyl and the benzoylamino moieties. The rationalized compounds (**8 a**–**l**) were first evaluated in silico to assess the ADMET and drug‐likeness profiles, synthesized using appropriate synthetic strategies and then tested in vitro under the National Cancer Institute DTP‐NCI60 program. The entire series exhibited potent anticancer activity against the renal cell carcinoma (RCC) cell line UO‐31, with compounds **8 c** and **8 g** effectively inhibiting cancer cell growth without excessive cytotoxic effects (growth percentages of −7 and −19, respectively). In silico induced fit docking (IFD) and molecular dynamics (MD) studies provided further insights into the putative mechanisms of action for both compounds, which were predicted to strongly bind key oncogenic proteins involved in RCC progression. The promising in vitro and in silico results herein presented provide a solid foundation for the development of a new series of small heterocyclic molecules with anticancer activity.

## Graphical abstract

A novel series of hybrid 4‐(4‐benzoylpiperazin‐1‐yl)‐6‐nitroquinoline‐3‐carbonitrile derivatives has been designed, synthetized and evaluated for anticancer properties on the NCI60 panel. Strong antiproliferative effects have been detected against renal cancer cells. In vitro results were supported by in silico ADMET, docking, and molecular dynamics studies, providing insights into their mechanisms of action.

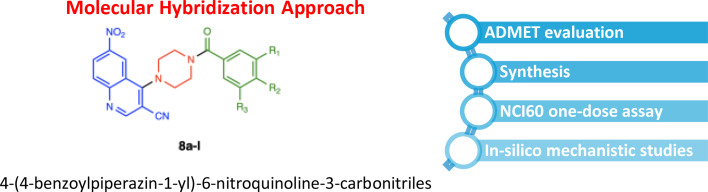

## Introduction

In the field of anticancer drug discovery, the quinoline motif stand out as one of the most interesting and widely studied nitrogen heterocycles due to its capability to provide both optimal pharmacokinetic and dynamic properties.[^1^, ^2^] Several quinoline derivatives, including those from natural sources, have demonstrated potent anticancer activity by targeting specific enzymes, receptors, and signaling pathways involved in cancer progression.[^3^, ^4^] Some of these agents have even received FDA approval, such as *cabozantinib*, *neratinib*, and *lenvatinib*, kinase inhibitors in the targeted cancer treatment.[^3^, ^4^]

Building on the success of quinoline derivatives, the six‐membered piperazine core is another preferred scaffold for anticancer drug development. Thanks to its chemical versatility and reactivity, as well as its unique physicochemical (e. g. solubility, basicity) and conformational properties (high flexibility), the introduction of the piperazine moiety in drug discovery campaigns has been successful and fruitful. This has led to the approval of numerous piperazine derivatives in anticancer therapy, such as *imatinib*, *olaparib* and *bosutinib*.[^5^, ^6^, ^7^]

**Figure 1:**
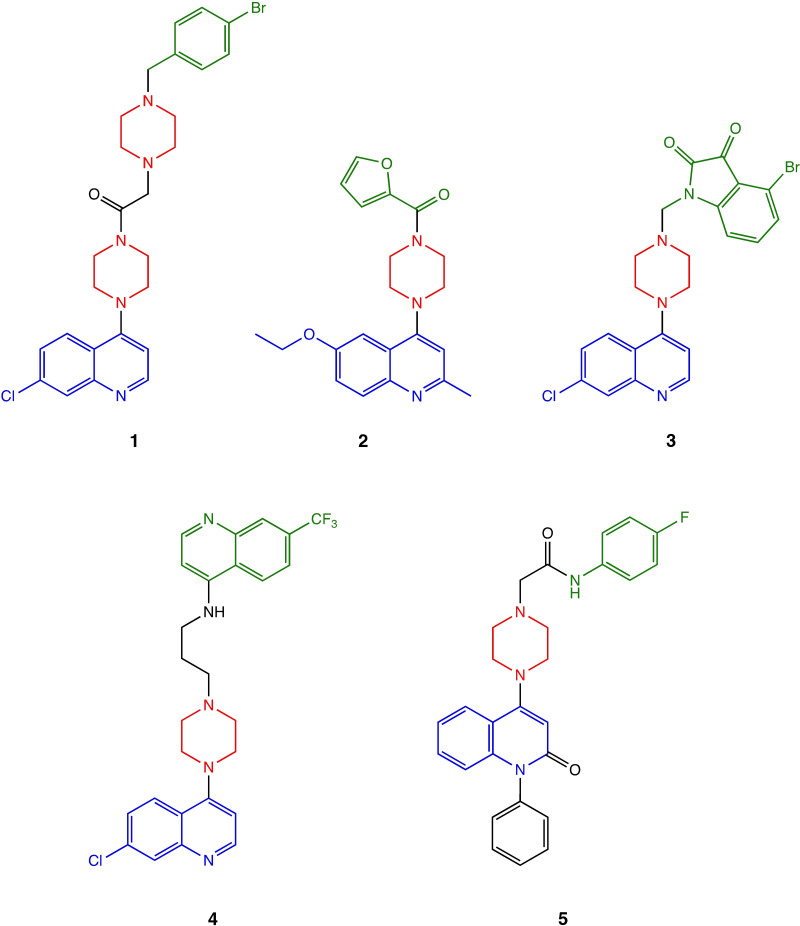
Chemical structure of several anticancer 4‐piperazinylquinoline hybrids 1–5 (quinoline, piperazine, and aromatic side moieties are shown in blue, red, and green, respectively).

The success of quinoline‐ and piperazine‐based drugs in recent decades underlines the importance of both pharmacophores in the design of new and more effective anticancer compounds. In this context, molecular hybridization–the combination two or more pharmacophoric fragments into a single molecular entity–is proving to be a promising strategy for optimizing lead structures.[^8^, ^9^, ^10^] As illustrated in Figure 1, several interesting piperazinyl‐quinoline hybrids from the literature exemplify this approach. In particular, the 4‐piperazinoquinoline core has contributed significantly to the medicinal chemistry library with numerous anticancer bioactive compounds.^[10]^ Compound **1**, with a quinoline scaffold, central piperazine motif as linker, and an aromatic side portion, exhibited a strong antiproliferative effect on the MCF‐7 and PC‐3 cell lines and a promising inhibitory activity against VEGFR‐2.^[11]^ Similarly, piperazinyl‐quinolines **2**–**5**, demonstrated noteworthy antiproliferative activities against various cancer cell lines.[^12^, ^13^, ^14^, ^15^]

**Figure 2:**
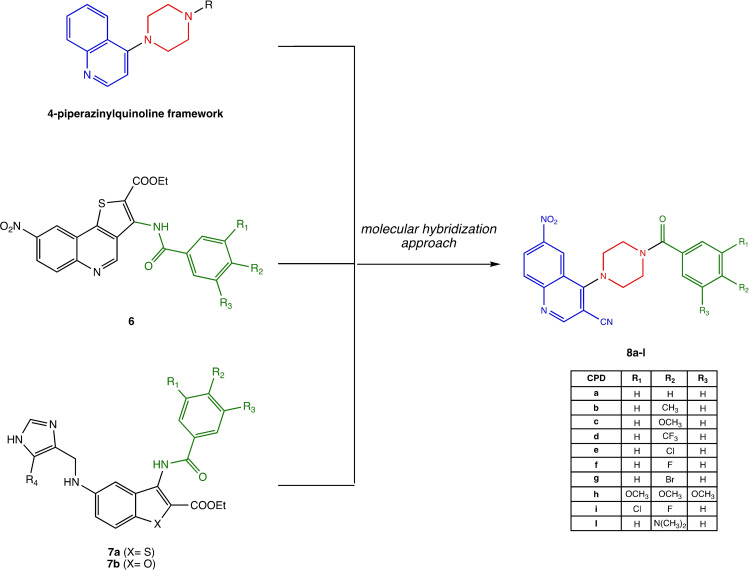
Chemical structure of the antiproliferative heterocyclic derivatives with a substituted benzoylamino moiety (in green) **6** and **7 a**, **b**, and of the newly developed 4‐(4‐benzoylpiperazin‐1‐yl)‐6‐nitroquinoline‐3‐carbonitrile compounds **8**, which are designed by a molecular hybridization strategy.

Based on this knowledge, in this work we have developed the new series of piperazinyl‐quinolines **8 a**–**l** with an aromatic side moiety as promising anticancer agents. The introduction of an aromatic benzoylamino motif on the second piperazine nitrogen, proved to be another interesting pharmacophoric portion. In previous studies, we have observed the importance of the benzoylamino motif in enhancing the antiproliferative activity in two different series of compounds of type **6** and **7 a**, **b**, (Figure 2).[^16^, ^17^, ^18^] The newly designed compounds **8 a**–**l** herein presented were firstly evaluated in silico for their drug‐likeness and ADMET properties, synthetized and evaluated in vitro for their antiproliferative activity. Molecular docking and dynamic studies were also employed to support the explanation of the potential mechanism of action of the most interesting compounds, as detailed in the following sections.

## Results and Discussion

### ADMET Properties Prediction

In drug discovery, the preliminary estimation of ADMET (Absorption, Distribution, Metabolism, Excretion, and Toxicity) and drug‐likeness parameters using in silico techniques is an invaluable aid to save both time and resources, increase the success rate, and consequently reduce the risks of failure in the preclinical and clinical phases.^[19]^

To gain insight into the drug‐likeness of our compounds, we decided to use the QikProp tool to calculate the #stars value.^[20]^ This parameter indicates the number of property or descriptor values that fall outside the 95 % range of similar values computed for known drugs. For all piperazinyl derivatives **8 a**–**l**, the obtained QikProp #stars value was 1 (Table 1), indicating promising pharmacokinetic and physicochemical properties of our compounds, considering the recommended QikProp value <5.

**Table 1:** Key predicted ADME properties calculated through SwissADME and QikProp tools.

CPD	QikProp Stars	QPPCaco2^[#]^	HOA%	P‐gp substrate	LRoF^[#]^	GV^[#]^	VV^[#]^	EV^[#]^	MV^[#]^	PAINS^[#]^
**8 a**	1	110.634	76.391	No	0	0	0	0	0	0
**8 b**	1	110.614	78.297	No	0	0	0	0	0	0
**8 c**	1	110.620	76.717	No	0	0	0	0	0	0
**8 d**	1	110.727	82.242	No	0	0	0	0	0	0
**8 e**	1	110.713	79.356	No	0	0	0	0	0	0
**8 f**	1	110.713	77.799	No	0	0	0	0	0	0
**8 g**	1	120.756	81.069	No	0	0	0	0	0	0
**8 h**	1	128.087	66.514	No	1	1	0	1	0	0
**8 i**	1	110.331	80.512	No	0	0	0	0	0	0
**8 l**	1	123.312	79.980	No	0	1	0	0	0	0

[#] Abbreviations: HOA %: predicted Human Oral Absorption on 0 to 100 % scale; QPP‐Caco2: predicted apparent Caco‐2 cell permeability in nm/sec; P‐gp, glycoprotein P; LRoF: Lipinski Rule of Five; GV: Ghose Violations; VV: Veber Violations; EV: Egan Violations; MV: Muegge Violations; PAINS: Pan‐Assay Interference Compounds.

**Figure 3:**
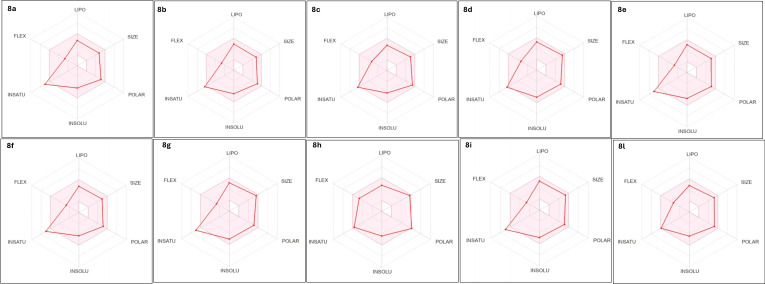
Bioavailability radar plot produced using the SwissADME tool calculations for compounds **8 a**–**l**.

In addition, the SwissADME tool was used to create a bioavailability radar plot for each compound, to allow a rapid and graphical appraisal of drug‐likeness, Figure 3. The hexagonal graph shows six axes representing six important properties for oral bioavailability: lipophilicity, size, polarity, solubility, fraction of saturated bonds, and flexibility. The range of optimal values for each parameter defines the pink area in the plot, and the radar plot for a molecule should fall almost entirely within this colored area (for further details about the parameters considered, see reference).^[21]^ The adequate oral bioavailability was predicted for most of the compounds analyzed. With the exception of the parameters fraction of saturated bonds and flexibility, which are higher and lower than the standard data, respectively, the values of the lipophilicity, size, polarity, solubility parameters are in the optimal range, Figure 3. This was also confirmed by the high values of Human Oral Absorption on a scale from 0 to 100 % (HOA%), (see Table 1, which gives an overview of the most interesting results, the complete matrices can be found in the Supporting Information SI‐1).

All compounds were predicted to be highly or completely absorbed in the gastrointestinal tract through a passive mechanism. This is evidenced by the apparent Caco‐2 cell permeability, which is a well‐recognized parameter for estimating the ability of xenobiotics to penetrate the gut‐blood barrier. Furthermore, the piperazinyl‐quinolines exhibited an optimal balance between lipophilicity and hydrophilicity (LogP and water solubility parameters are listed in the Supporting Information, Table SI‐1).

**Figure 4:**
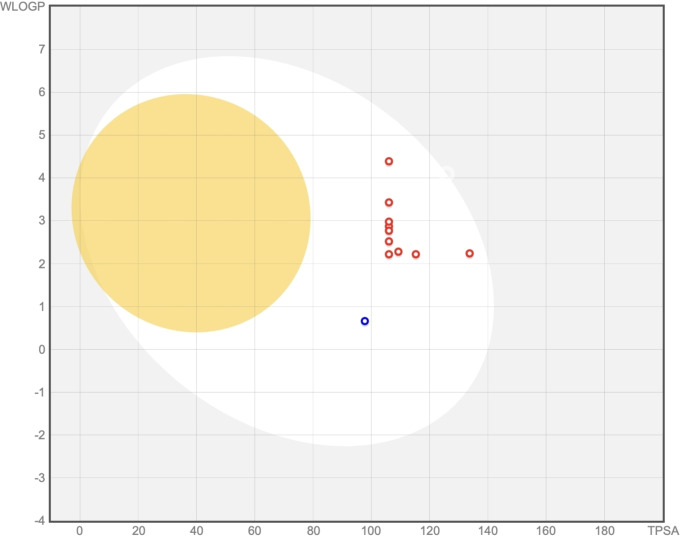
BOILED‐Egg graph provided by the SwissADME tool for compound **8 a**–**l** indicates the following: compounds falling within the grey region are predicted to neither be passively absorbed in the gastrointestinal tract; those in the white region are expected to be passively absorbed in the intestine; compounds in the yellow (yolk) region are expected to pass the blood‐brain barrier (BBB). The red dots refer to compounds that are not substrates of Pgp.

Derivatives **8 a**–**l** do not appear to be substrates of P‐gp (Table 1), one of the best characterized efflux pumps that frequently contributes to suboptimal bioavailability and drug resistance in cancer cells (active efflux mechanisms). All data predicted for the investigated piperazinyl‐quinolines are plotted and summarized in an intuitive graphical classification model called BOILED‐Egg, Figure 4 (see refs.[^21^, ^22^] for more details).

Finally, drug‐likeness and medicinal chemistry filters commonly used in virtual studies to screen compound libraries were applied, including the Lipinski, Ghose, Veber, Egan, and Muegge rules, and the PAINS filter, Table 1. None of the compounds showed more than one violation of the rules considered, confirming their drug‐likeness. All piperazinyl‐quinolines were free of PAINS groups, indicating no potential promiscuity in biological assays.

### Synthesis of Benzoylpiperazynil‐Quinolines 8 a–l

**Scheme 1:**
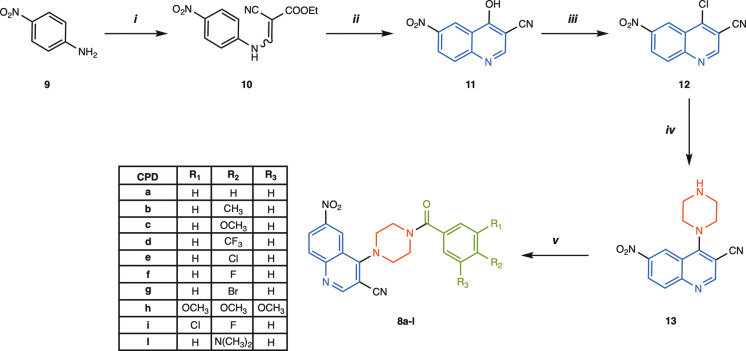
Synthesis of 6‐nitro‐4‐piperazinylquinoline **8 a**–**l** derivatives. Reagents and conditions: (i) ethyl‐2‐cyano‐3‐ethoxyacrylate, toluene, reflux 12 h; (ii) Dowtherm A, N_2_, reflux, 10 h; (iii) POCl_3_, reflux, 8 h; (iv) anhydrous piperazine, MeCN, rt, 1 h (v) appropriate benzoyl chloride (1.25 eq.), DIPEA (1.5 eq.), dry DCM, rt, 24 h.

The promising ADMET prediction for the 4‐(4‐benzoylpiperazin‐1‐yl)‐6‐nitroquinoline‐3‐carbonitrile derivatives **8 a**–**l** encouraged their synthesis. Scheme 1 shows the overall preparation pathway. In the first three steps, the quinoline core was synthesized, and subsequently coupled with the piperazine moiety. Finally, various substituted benzoyl chlorides were used to introduce the aromatic side fragment.

The Gould‐Jacobs approach was chosen for the synthesis of the quinoline nucleus,^[23]^ as it has proven successful in the preparation of the key 4‐hydroxyquinoline intermediates. In particular, the reaction of p‐nitroaniline **9** with ethyl‐2‐cyano‐3‐ethoxyacrylate gave the intermediate **10** as a mixture of E/Z regioisomers in quantitative yields within a relatively short reaction time (12 h). According to the Gould‐Jacobs protocol, the 4‐hydroxy‐6‐nitroquinoline‐3‐carbonitrile intermediate **11** was obtained by intramolecular thermal cyclization in excellent yield (85 %). The reaction was carried out by prolonged heating at temperatures between 257–260 °C, using high‐boiling solvents such as diphenyl ether or Dowtherm. Subsequent aromatic chlorination with POCl_3_ produced intermediate **12**, featuring an optimal leaving group at the C4 position. These results are consistent with the literature.[^16^, ^24^]

After the synthesis of the quinoline scaffold, the piperazine linker was introduced at the C4 position by nucleophilic aromatic substitution (SnAr). The presence of two electron‐withdrawing groups, 3‐CN and 6‐NO_2_, significantly activated the chlorine for the nucleophilic displacement by the piperazine nitrogen, which was added in stoichiometric amount. As a result, the desired 4‐piperazinylquinoline intermediate **13** was obtained in quantitative yield at room temperature in 1 h, without the need for a base and used without further purification.

In the final step, the aromatic side fragment was introduced by nucleophilic acyl substitution of the second piperazine nitrogen with variously substituted benzoyl chlorides. This reaction was carried out under strictly anhydrous conditions, using dry dichloromethane (DCM) as solvent and N,N‐diisopropylethylamine (DIPEA) as base, to give the title 4‐(4‐benzoylpiperazin‐1‐yl)‐6‐nitroquinoline‐3‐carbonitrile derivatives **8 a**–**l** in good to optimal yields (65–95 %).

### Biological Assays: One‐Dose NCI60 Human Tumor Cell Lines Screen

The NCI‐60 Human Tumor Cell Lines Screen has been a cornerstone of cancer research worldwide. It uses 60 different human tumor cell lines divided into nine panels (leukemia, melanoma, and cancers of the lung, colon, brain, ovary, breast, prostate, and kidney) to identify and characterize novel compounds with potential tumor‐inhibiting or tumor‐killing properties.[^25^, ^26^] To comprehensively evaluate the anticancer potential of the derivatives presented in this study, all synthesized compounds were submitted to the NCI. Following the specific guidelines for compound selection described in the Experimental Section, the NCI accepted the entire set of compounds, including intermediate **13**, for preliminary evaluation in the one‐dose assay.^[27]^

Each compound was tested at the single concentration of 10^−5^ M (10 μM) against the full NCI60 panel. The results were expressed as a percentage of growth (G %) compared to untreated control cells, which is a clear indicator of the anticancer potential of each compound. A G %>100 indicates no effect on the proliferation of cancer cell (inactive), while values in the range 0 to 100‐G % reflect varying degrees of growth inhibition. G %<0 means cytotoxicity, i. e. cell death. The full G % data for the eleven compounds tested are provided in the Supporting Information (Table SI‐2).

Significant antiproliferative effects were observed within the renal cancer panel, particularly against the renal cell carcinoma (RCC) cell line UO‐31. As detailed in Table 2, a mean G % value of −9.1 was calculated, indicating a strong antiproliferative effect. In general, the introduction of the aromatic benzoylamino side moiety was useful for the biological activity: seven out of ten final compounds exhibited a significantly improved G % value compared to the intermediate **13**. Compounds **8 b** (4’−CH_3_), **8 e** (4’−Cl), **8 l** (4’−N(CH_3_)), and **8 h** (3’, 4’, 5’−OCH_3_) were the most cytotoxic, with G % values below −40. Interestingly, derivatives **8 c** (4’−OCH_3_) and **8 g** (4’−Br) were the most promising, showing a more balanced antiproliferative/cytotoxic profile, with G % values of −6.68 and −19.40, respectively. On the other hand, only three compounds, **8 a** (4’−H), **8 d** (4’−CF_3_), and **8 i** (3’−Cl, 4’−F) exhibited a modest inhibition of cell growth, with a G % comparable to **13** (>60).

**Table 2:** G % values determined for the **13** and **8 a**–**l** compounds against renal carcinoma cell line UO‐31 in the one‐dose assay (10^−5^ M).

Compound	NSC Number	G %
13	847727	62.52
**8 a**	847732	69.49
**8 b**	847728	−70.71
**8 c**	847729	−6.68
**8 d**	847730	61.18
**8 e**	847731	−69.88
**8 f**	847733	−54.57
**8 g**	847911	−19.40
**8 h**	847736	−89.02
**8 i**	847735	63.37
**8 l**	847734	−46.38
Mean G %		−9.10

Special attention should also be paid to compound **8 g**, which features a 4’−Br substituent on the side phenyl fragment. Unlike the other analogues, **8 g** showed a significantly promising mean G % of 50.85, demonstrating an overall remarkable antiproliferative profile with low to weak cytotoxic effects, especially against highly aggressive cancer types. Supporting Figure SI‐3 illustrates the one‐dose mean graph for **8 g** provided by the NCI, whereas the detailed results for cell lines where a growth percentage (G %) of less than 55 % was observed are reported in Table 3.

**Table 3:** Growth Percentage (G %) values of compound **8 g** across NCI‐60 cell lines with values below the 55 % threshold.

Panel	Cell Line	G %
Leukemia	K‐562	32.49
	MOLT‐4	32.88
	SR	40.76
NSCLC	HOP‐62	41.26
	HOP‐92	−3.39
	NCI−H226	36.22
	NCI−H460	40.65
	NCI−H522	26.26
COLON CANCER	HCT‐116	48.7
CNS CANCER	SF‐268	49.67
	SF‐295	42.6
	SF‐539	47.69
	SNB‐19	54.69
	SNB‐75	4.43
	U251	47.86
MELANOMA	SK‐MEL‐2	54.57
	SK‐MEL‐5	45.79
OVARIAN CANCER	OVCAR‐4	41.22
	SK‐OV‐3	54
RENAL CANCER	786‐0	47.95
	A498	25.97
	CAKI‐1	31.87
	RXF‐393	32.82
	UO‐31	−19.4
BREAST CANCER	MDA‐MB‐231/ATCC	42.43
	HS‐578T	7.71
	BT‐549	39.87
	T‐47D	35.06
	MDA‐MB‐468	35.96

As depicted, notable growth inhibition values were observed within the leukemia panel, where three cell lines (K‐562, MOLT‐4, and SR) had G % values lower than the mean (32.49, 32.88, and 40.76, respectively). Similar results were seen in five out of nine cell lines in the NSCLC panel, which is known for its aggressiveness and treatment issues. Particularly, **8 g** was able to completely halt the growth of the HOP‐92 cell line, and achieve a G % around 0, indicating that no net growth occurred during the experiment.

Significantly, nearly complete growth inhibition was observed against the SNB‐75 cell line from the CNS panel (G %=4.43), which is associated with an aggressive glioblastoma cancer type. Within the renal cancer panel, besides the UO‐31 cell line, promising G % values below 40 were obtained for A498, CAKI‐1, and RXF‐393 (25.97, 31.87, and 32.82, respectively). Special mention should also be made of the breast cancer panel, where an outstanding mean G % value of 36 was computed, particularly against the multidrug‐resistant TNBC cell line HS 578T (G %=7.71).

Generally, none of the derivatives exhibited significant overall growth inhibition activities (mean G %>90), with the exception of **8 g**, as discussed. Consequently, none of the compounds qualified for the five‐dose assay, which is used to determine the IC_50_.

### In Silico Mechanicistic Insights: Structure‐Based Studies on Selected RCC Targets

#### Induced Fit Docking (IFD) Studies

In light of the results presented, in silico structure‐based techniques were used to elucidate the potential mechanism of action and biological targets responsible for the anticancer activity observed in the renal cell carcinoma. The analysis focused specifically on compounds **8 c** and **8 g**, which demonstrated the most promising biological activity.

A comprehensive investigation of the molecular biology of RCC has allowed us to identify numerous deregulated/mutated proteins closely associated with the development and progression of the disease. In particular, key transmembrane growth factor receptors such as EGFR, VEGFR, PDGFR, FGFR, c‐MET, c‐KIT, RET, ALK‐1, AXL, and PD−L1 are highly upregulated in RCC, along with intracellular downstream effectors like the PI3 K/Akt/mTOR and Ras/Raf/MAPK cascades.[^28^, ^29^, ^30^, ^31^] Additionally, anti‐apoptotic proteins such as BCL‐2 and BCL‐XL, along with epigenetic modulators like histone lysine‐specific demethylase 1 (LSD1), are specific markers overexpressed in RCC.[^28^, ^29^, ^30^, ^31^]

For these reasons, induced fit molecular docking calculations (IFD) were performed against the most relevant and druggable targets mentioned above. A total of 19 biological target X‐ray structures were selected, retrieved from the Protein Data Bank[^32^, ^33^] and prepared for the molecular modelling studies (details are reported in the Experimental section). These targets include EGFR (PDB id: 1 M17);^[34]^ VEGFR‐2 (PDB id: 3WZD);^[35]^ RET (PDB id: 6NEC);^[36]^ c‐KIT (PDB id: 6GQK);^[37]^ c‐MET (PDB id: 3LQ8);^[38]^ AXL (PDB id: 7DXL);^[39]^ PDGFRA (PDB id: 6JOL);^[40]^ FGFR1 (PDB id: 4F63);^[41]^ PDL1 (PDB id: 5 N2D);^[42]^ ALK1 (PDB id: 3MY0);^[43]^ PI3Kα (PDB id: 7 K6 M);^[44]^ PI3Kδ (PDB id: 4XE0);^[45]^ AKT/PKB (PDB id: 3OCB);^[46]^ mTOR (PDB id: 4JSX);^[47]^ B‐RAF (PDB id: 1UWH);^[48]^ C‐RAF (PDB id: 3OMV);^[49]^ BCL‐2 (PDB id: 6QGK);^[50]^ Bcl‐XL (PDB id: 3QKD);^[51]^ LSD1 (PDB id: 5LGN).^[52]^

Table 4 provides an overview of the in silico results (IFD scores) obtained for the best docked pose of compounds **8 c** and **8 g** compared to the reference inhibitors (corresponding co‐crystallized ligands) against all the selected biological targets (the full output matrix is given in the Supporting Information SI‐4, whereas the most interesting 2D ligand‐protein interaction maps are reported in SI‐5)). From an in‐depth analysis, it was observed that for 4 out of the 19 targets considered (AXL, C‐RAF, BCL‐2, LSD1), the newly designed compounds exhibited IFD scores in the same range or higher than the control inhibitors. This indicates a potentially strong binding affinity and favorable binding modes of both compounds to these targets, suggesting their promising activity as inhibitors as well as specificity against these proteins. On the other hand, only limited interactions were detected for the other proteins studied.

**Table 4:** Induced Fit Docking (IFD) scores for the best docked pose of compounds **8 c** and **8 g** against the selected targets involved in RCC. The IFD scores for reference ligands (known inhibitors) are reported for comparison.

Protein	Compounds **8 c**, **g**	Refs.^[#]^
EGFR	−654.46÷−654.55	−665.26
VEGFR‐2	−603.38÷−602.79	−611.44
RET	−661.08÷−662.14	−665.93
c‐KIT	−663.09÷−663.92	−673.74
c‐MET	−592.92÷−594.76	−606.71
AXL^[^*^]^	−568.87÷−573.63	−571.11
PDGFRA	−600.00÷−600.27	−610.41
FGFR1	−612.94÷−615.05	−626.23
PDL1	−560.26÷−562.29	−567.20
ALK1	−654.96÷−657.58	−659.74
PI3 Kα	−1996.10÷−1996.13	−2010.69
PI3 Kδ	−1760.24÷−1760.70	−1763.04
AKT/PKB	−731.49÷−731.66	−736.79
mTOR	−2325.47÷−2327.08	−2332.93
B‐RAF	−572.16÷−572.35	−580.15
C‐RAF^[^*^]^	−549.25÷−551.26	−551.35
BCL‐2^[^*^]^	−290.32÷−291.76	−293.35
Bcl‐XL	−333.86÷−335.07	−350.00
LSD1^[^*^]^	−1442.86÷−1444.67	−1443.16

[*] Target proteins for which **8 c** and **8 g** have reported IFD Score value comparable to/higher than that of the reference compounds. [#] Corresponding co‐crystallized inhibitors were used as reference compounds.

The detailed results of the Induced Fit Docking (IFD) studies, including IFD scores, Glide scores, and Prime Energy scores for the best docked poses of **8 c** and **8 g** towards the four best‐ranked targets (AXL, C‐RAF, BCL‐2, LSD1), are reported in Table 5. A thorough analysis of the results shows that at least one of the investigated compounds (**8 c** or **8 g**) exhibited higher values for AXL and LSD1 than the reference inhibitor for all three calculated parameters. Therefore, these compounds could potentially have stronger binding affinities and better interaction profiles with AXL and LSD1 compared to the known inhibitors.

**Table 5:** Detailed view of IFD, Glide and Prime Energy scores for compounds **8 c** and **8 g** retrieved from Induced Fit Docking simulation in comparison with the reference co‐crystallized inhibitors.

CPD	AXL			C‐RAF			BCL‐2			LSD1		
	IFD score	Glide score	Prime Energy	IFD score	Glide score	Prime Energy	IFD score	Glide score	Prime Energy	IFD score	Glide score	Prime Energy
**8 c**	−573.63	−11.152	−11249.52	−551.26	−12.243	−10780.43	−290.32	−7.620	−5654.03	−1444.67	−8.839	−28716.54
**8 g**	−568.87	−9.674	−11184.01	−549.25	−11.329	−10758.40	−291.76	−8.392	−5667.29	−1442.86	−9.124	−28674.74
Ref.^[^*^]^	−571.11	−11.005	−11202.06	−551.35	−11.075	−10805.54	−293.35	−7.977	−5707.50	−1443.16	−8.460	−28694.01

[*] The reference inhibitors considered were the corresponding co‐crystallized ligands: CID156613395 (AXL), CID11653652 (C‐RAF), CID138393395 (BCL‐2), CID51049245 (LSD1).

With respect to the C‐RAF and BCL‐2 targets, the IFD scores for the same compounds were similar to those of the reference ligands, while their Glide and Prime Energy scores were either better or comparable.

An in‐depth analysis of the literature reveals that numerous quinoline‐based compounds have been identified as inhibitors of these targets.[^3^, ^4^, ^53^, ^54^, ^55^] These findings support our hypothesis that compounds **8 c** and **8 g**, with the key pharmacophoric quinoline moiety, may exert anticancer effects by modulating these proteins.

All the best ranked ligand‐protein complexes were submitted to molecular dynamics (MD) simulation studies in order to investigate the binding affinity, the stability and the strength of the interactions within the binding pockets along time.

#### Molecular Dynamics Simulations

For molecular dynamic studies Desmond software from the Schrödinger suite was used in explicit solvent mode (water).^[56]^ All‐atom molecular dynamics (MD) simulations were performed for the best‐docked pose from induced fit docking (IFD) studies for each of the twelve ligand‐protein complexes (**8 c** and **8 g** with AXL, C‐RAF, BCL‐2, and LSD1, along with the reference inhibitors bound to their respective targets) over a 100‐nanosecond timescale. The Newtonian dynamics of each system placed in an appropriate thermodynamic environment (isothermal‐isobaric) was simulated, producing a series of snapshots or frames that capture coordinates, velocities, and energies of the particles. The trajectories generated were analyzed statistically to extract various properties of the model systems investigated, as reported in the following paragraphs (further parameters details selected for the simulation are reported in the Experimental section).

#### Ligand‐Protein Complexes Stability Analysis

To first evaluate the dynamic stability of the simulated ligand‐protein bound co‐systems, the trajectories generated were visually inspected. This examination revealed that all complexes remained stable throughout the entire simulation period. No significant structural distortions, large‐scale domain movements in the protein, as well as ligand displacement from the binding pocket were detected.

A more specific analysis was then performed by computing several well recognized geometric properties, such as the root‐mean‐square deviation/displacement (RMSD), which measures the displacement of selected atoms relative to the initial frame of the simulation (t=0). This parameter allows to track modification in structural conformation of the protein and ligand during the equilibration process. As general consideration, a stable RMSD mean value (convergence) with minor fluctuations (1 to 3 Å) indicates equilibration and stability of the system, while larger fluctuations suggest potential conformational changes and insufficient equilibration. In this paper, the analysis was conducted on the most commonly measured RMSD values, referred to the Cα protein atoms (backbone) and to the ligand heavy atoms when the protein‐ligand complex is first aligned on the protein backbone of the reference time.

**Figure 5:**
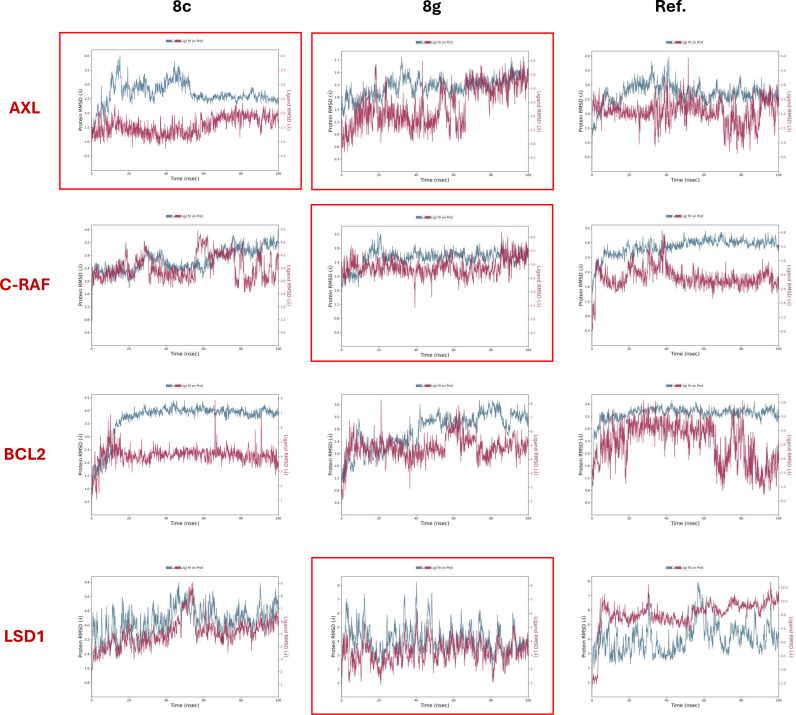
Protein backbone and ligand RMSD (expressed in Ångstroms) plots for the trajectories of the twelve simulated complexes over a 100 ns timescale. Each plot features two Y‐axes: the left Y‐axis indicates the RMSD scale for the protein backbone (Cα atoms), and the right Y‐axis corresponds to the RMSD scale for the ligand heavy atoms. The X‐axis represents the simulation time in nanoseconds. The blue curves show the RMSD of the protein backbone (Cα atoms), while the red curves display the RMSD of the ligand heavy atoms aligned to the protein backbone. Red boxes highlight simulations where the RMSD values are optimized relative to those of the reference inhibitors.

In light of these general considerations, in Figure 5 the plots showing the trend of both protein backbone (Cα) and ligand heavy atoms RMSD during the whole 100 ns simulation time are reported for the twelve complexes simulated.

Both compounds **8 c** and **8 g** demonstrated notable stability when complexed with AXL kinase. They reached equilibrium after approximately 60 ns of simulation, with RMSD values for both the ligand and the protein fluctuating in the average range of 2–3 Å. Additionally, in the latter part of the simulation, both compounds showed better convergence and fewer fluctuations than the reference ligand, which in turn did not appear to be equilibrated. This indicates improved stability and better equilibration under the simulated conditions.

Similarly, the complex **8 g**/C‐RAF showed better stability than the reference inhibitor, since it reached the equilibrium and the convergence almost at the beginning of the simulation (after 15 ns), displaying narrow fluctuations and average RMSD values in the acceptable range of 2.5–3.5 Å.

Neither of the two studied compounds exhibited acceptable stability in complex with BCL‐2 in the time frame considered, as evidenced by both the high and distant RMSD values for protein and ligands, and the excessive fluctuations.

Interestingly, special attention should be given to the MD runs of LSD1, where, at first glance, the RMSD values appear to be higher than the standard acceptable ranges. However, the 3D structure of LSD1, which features a helix‐turn‐helix elongated motif extending from the spherical core,^[57]^ could be responsible of increased mobility and fluctuations, particularly in regions far from the binding sites. Taking this into account, mean RMSD values higher than 3 Å could be accepted in this case. In this light the complex of LSD1 with **8 g** seems to reach a better equilibrium than the reference ligand, with better convergence and less fluctuations, especially in the last part of the simulation. In particular, the average RMSD values for the **8 g**‐LSD1 complex were very close and both in the range 3–6 Å after the equilibration. On the other hand, these values were higher for the reference inhibitors, with average ligand RMSD significantly higher than protein backbone RMSD (in the range 9–12 Å and 4–7 Å, respectively).

In the light of these considerations, compound **8 c** is predicted to have potential strong stability in the complexation with AXL. On the other hand, compound **8 g** seemed to have high stability when complexed with AXL, C‐RAF and LSD1, thus with a potential multitarget profile (in Figure 5 the plots referred to the best simulations are highlighted through a red box). Considering the importance of these targets not only in renal cancer pathogenesis but also in many other hyperproliferative malignancies,[^53^, ^57^, ^58^, ^59^, ^60^] these intriguing results could partly explain why compound **8 g** exhibited a broader antiproliferative activity across the NCI60 panel compared to **8 c**, especially against several lung, CNS, and breast cancer cell lines. This different behavior could be ascribed to the different steric hindrance and electronic feature of 4’−OCH_3_ and 4’−Br group, with the latter being able of occupying more the space into the protein binding sites and forming also halogen bonds.

**Figure 6:**
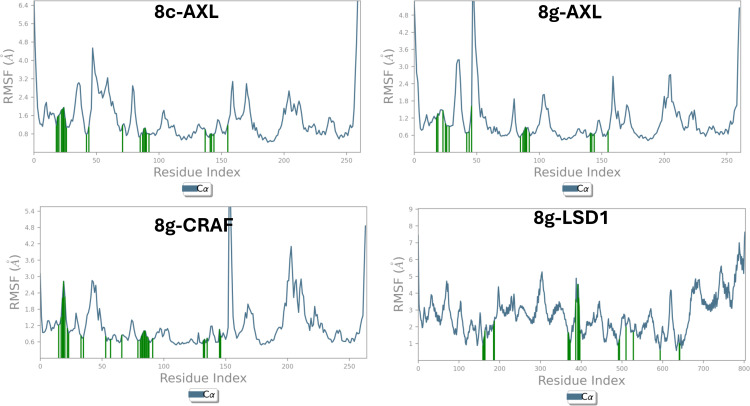
Graphical representation of the root mean square fluctuations (RMSF) of the protein Cα atoms for the four best‐performing ligand‐protein complexes: **8 c**‐AXL, **8 g**‐AXL, **8 g**‐CRAF, **8 g**‐LSD1. The X‐axis shows the residue index, while the right Y‐axis displays the RMSF values in Ångstroms. The vertical green bars indicate residues within the catalytic pocket that are involved in ligand binding.

For these reasons, these four simulations were selected for further analysis to better estimate the stability of the selected complexes. The mobility and flexibility of protein residues following ligand binding were evaluated by computing the root mean square fluctuation (RMSF), and the results are plotted in Figure 6. RMSF measures the local conformational changes of protein residues induced by ligand binding as function of protein residues. Low RMSF values indicate limited flexibility and mobility, which are typical for the more structured and rigid parts of the protein, such as the α‐helices and β‐sheets within the binding pocket, especially when these regions are involved in strong interactions with the ligand. In contrast, high RMSF values are expected for the N/C terminal ends and loops, which are more flexible and freer to fluctuate in 3D space. This analysis helps in understanding how ligand binding affects the stability and dynamics of different protein regions. As shown in Figure 6, the complexes of ligand **8 c** to AXL, as well as ligand **8 g** to AXL, C‐RAF, and LSD1, demonstrated stability comparable to or better than the reference inhibitors, as indicated by the low RMSF values, particularly for residues within the binding pocket involved in ligand interactions (RMSF<3 Å). Higher RMSF values were observed for regions distant from the binding site and not involved in the catalytic domain, reflecting their greater flexibility. A similar trend was observed for the control ligands (RMSF plots for reference inhibitors are available in the Supporting Information SI‐6).

#### Ligand‐Protein Interaction Pattern Analysis

To gain a more detailed understanding of the molecular factors contributing to the stability and strength of the complexes, the ligand‐protein interaction patterns were analyzed by integrating information from both Induced Fit Docking (IFD) and Molecular Dynamics (MD) studies. By combining these two methods, it was possible to better elucidate the dynamic nature of ligand binding, accounting for both the flexibility of the protein and ligand and the dynamic environment of the binding site.

**Figure 7:**
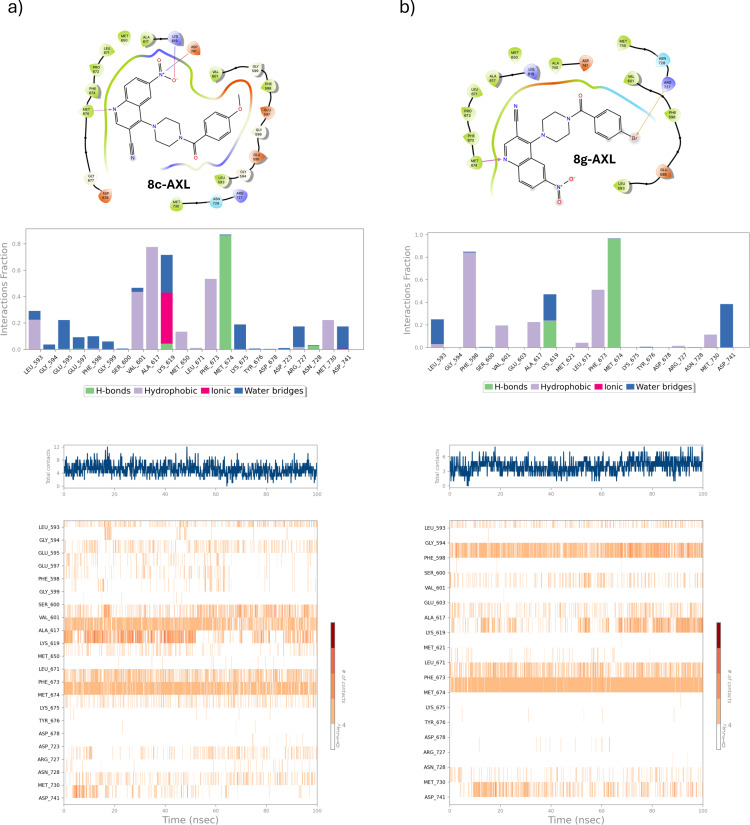
Ligand‐protein interaction analysis for the complexes of both **8 c** (panel a) and **8 g** (panel b) with AXL. Hydrogen bonds, hydrophobic interactions, ionic interactions, water bridges and halogen bonds were considered as contributes. For each complex, the 2D ligand interaction diagram, the average fraction of simulation time per protein residue interacting with the ligand, the total number of interactions over time, and the total number of interactions formed across the trajectory by each specific residue are reported.

**Figure 8:**
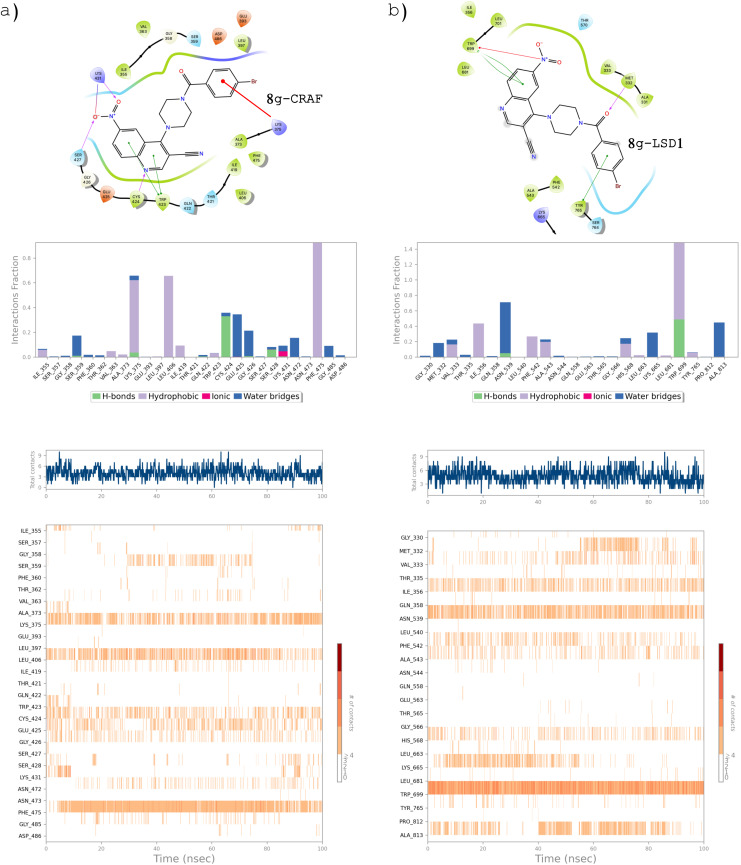
Ligand‐protein interaction analysis for the complexes of both **8 g** with C‐RAF and LSD1, respectively. Hydrogen bonds, hydrophobic interactions, ionic interactions, water bridges and halogen bonds were considered as contributes. For each complex, the 2D ligand interaction diagram, the average fraction of simulation time per protein residue interacting with the ligand, the total number of interactions over time, and the total number of interactions formed across the simulation by each specific residue are reported.

Figures 7 and 8 present the 2D ligand‐protein interaction diagrams and other relevant plots for the four best complexes identified. These figures provide both qualitative and quantitative insights into the types and numbers of contacts between protein residues and the ligand throughout the entire trajectory. For this study, all available interaction types–hydrogen bonds, hydrophobic interactions, ionic interactions, water bridges, and halogen bonds–were considered. Notably, both compounds **8 c** and **8 g** were capable of strongly interacting with many crucial residues within the AXL kinase catalytic domain, with most of the persistent contacts being hydrogen bonds, water bridges and hydrophobic interactions (e. g.: Phe^598^, Val^601^, Ala^617^, Lys^619^, Phe^673^, Met^674^ and Asp^741^).

In both cases, the total number of contacts remained approximately stable throughout most of the simulation time, indicating that equilibrium had been reached.

Similar consideration can be done for the complexes **8 g**‐CRAF and **8 g**‐LSD1 (Figure 8). In the former, many hydrophobic interactions and water mediated H‐bonds (bridges) occurred between the aromatic portion of the ligand (quinoline and benzoylamino moieties) and key residues as Lys^375^, Leu^406^, Trp^423^, Cys^424^ and Phe^475^ and persisted for most of the simulation.

Analogously, even in complex with LSD1 **8 g** was capable to well occupy the catalytic cleft, interacting mainly through hydrophobic residues, as Val^333^, Ile^356^, Lys^665^, and, particularly, Trp^699^.

As a general observation, the quinoline ring, due to its electron‐deficient nature and the presence of a hydrogen bond acceptor in the heterocyclic nitrogen, plays a crucial role in forming most of the stabilizing interactions. In contrast, the benzoylamino portion is primarily responsible for forming hydrophobic contacts. Regarding the para‐substitution, the different behavior between the 4′‐methoxy and 4′‐bromo derivatives can be attributed to the distinct steric hindrance and electronic properties of the 4−OCH_3_ and 4−Br groups, with the latter being able to occupy more space within the protein binding sites and form halogen bonds.

## Experimental Section

### Chemistry

#### General Information

Unless specified otherwise, all reagents and solvents were obtained from commercial suppliers and used without additional purification. Melting points were measured on a Büchi Tottoli capillary apparatus and are uncorrected. The ^1^H NMR and ^13^C NMR spectra were acquired at 400 MHz and 100 MHz, respectively, in CDCl_3_ or DMSO‐d_6_ using a Bruker AC−E series 400 MHz spectrometer. Chemical shift values are reported in parts per million (ppm) with tetramethylsilane (TMS) as the internal standard. The following abbreviations are used: br s (broad signal), s (singlet), d (doublet), t (triplet), q (quartet), m (multiplet), and rt (room temperature). The purity of all compounds tested in biological assays was confirmed to be greater than 95 % by high‐performance liquid chromatography/mass spectrometry (HPLC/MS) analysis. Mass spectrometry was performed using an Agilent 6540 UHD accurate‐mass quadrupole time‐of‐flight (Q‐TOF) spectrometer. Microanalytical data were consistent with theoretical values within ±0.4 %. Thin‐layer chromatography (TLC) was conducted on precoated silica gel GF254 plates, and compounds were visualized using a UV lamp at 254 nm. Column chromatography was carried out using Merck silica gel (230 and 400 mesh) or a Biotage FLASH40i chromatography system with prepacked cartridges. 4‐nitroaniline (**9**) is commercially available. The intermediate **10**–**12** were obtained following procedures reported in the literature.[^16^, ^24^]

#### Experimental Procedures and Product Characterization

##### 6‐Nitro‐4‐(Piperazin‐1‐yl)quinoline‐3‐Carbonitrile (13)

To a stirring mixture of 4‐chloro‐6‐nitroquinolin‐3‐carbonitrile **12** (400 mg, 1.7 mmol) in acetonitrile, piperazine (147.5 mg, 1.78 mmol) was added and the mixture was allowed to stir at RT for 2 h. The solvent was then evaporated in vacuo, the residue was resuspended in saturated solution of Na_2_CO_3_ and extracted with DCM (3×25 mL). The organic phase was dried over sodium sulphate, filtered and concentrated to give 6‐nitro‐4‐(piperazin‐1‐yl) quinoline‐3‐carbonitrile as yellow powder. Quantitative yield. Mp 160–162 °C. ^1^H NMR (CDCl_3_) δ: 3.20–3.27 (m, 4H, 2×CH_2_), 3.77–3.84 (m, 4H, 2×CH_2_), 8.15 (d, 1H, J=9.2 Hz, H‐8), 8.49 (dd, 1H, J=9.2, 2.5 Hz, H‐7), 8.83 (s, 1H, H‐2), 9.00 (dd, 1H, J=2.5, 0.5 Hz, H‐5). ^13^C NMR (CDCl_3_) δ: 46.6 (CH_2_), 54.5 (CH_2_), 96.9, 117.7, 122.0, 122.1 (CH), 125.0 (CH), 132.2 (CH), 145.0, 152.8, 155.8 (CH), 160.5. HRMS‐ESI [(M+H)^+^]: m/z calculated for C_14_H_13_N_5_O_2_: 284.1142; found: 284.1144.

#### General Procedure for the Synthesis of 4‐(4‐Benzoylpiperazin‐1‐yl)‐6‐Nitroquinoline‐3‐Carbonitrile Derivatives 8

To a stirred solution of **13** (1 eq.) in dry DCM was added the DIPEA (1.5 eq.) at room temperature. At the resulted mixture, cooled at 0 °C, the appropriate benzoyl chloride (1.25 eq.) was slowly added. The reaction mixture was allowed to stir at room temperature (25 °C) for 24 h, then the crude was washed with HCl 1 N and extracted with DCM; the organic layer was dried over sodium sulphate and evaporated under vacuum. The precipitate was purified by chromatography column using petroleum ether/EtOAc 2 : 1 in gradient as eluant. Recrystallized from Et_2_O.

#### 4‐(4‐Benzoylpiperazin‐1‐yl)‐6‐Nitroquinoline‐3‐Carbonitrile (8 a)

Yield 71 %. Mp 179–180 °C. ^1^H NMR (CDCl_3_) δ: 3.61–4.30 (br m, 8H, 4×CH_2_), 7.42–7.54 (m, 5H, H‐2’, H‐3’, H‐4’, H‐5’, H‐6’), 8.21 (d, 1H, J=9.1 Hz, H‐8), 8.53 (dd, 1H, J=9.2, 2.5 Hz, H‐7), 8.89 (s, 1H, H‐2), 8.97 (d, 1H, J=2.5 Hz, H‐5). ^13^C NMR (CDCl_3_) δ: 53.1(CH_2_), 98.0, 117.3, 121.5(CH), 122.1, 125.3 (CH), 127.3 (CH), 128.8 (CH), 130.4 (CH), 132.6 (CH), 134.8, 145.4, 152.7, 155.5 (CH), 160.2, 170.8. HRMS‐ESI [(M+H)^+^]: m/z calculated for C_21_H_17_N_5_O_3_: 388.1404; found: 388.1407.

##### 4‐(4‐(4‐Methylbenzoyl)piperazin‐1‐yl)‐6‐Nitroquinoline‐3‐Carbonitrile (8 b)

Yield 91 %. Mp 166–168 °C. ^1^H NMR (DMSO‐d_6_) δ: 2.35 (s, 3H, CH_3_), 3.83 (br s, 8H, 4×CH_2_), 7.29 (d, 2H, J=8.0 Hz, H‐3’, H‐5’), 7.40 (d, 2H, J=8.0 Hz, H‐2’, H‐6’), 8.15 (d, 1H, J=9.2 Hz, H‐8), 8.51 (dd, 1H, J=9.2, 2.5 Hz, H‐7), 8.90 (d, 1H, J=2.5 Hz, H‐5), 8.95 (s, 1H, H‐2). ^13^C NMR (DMSO‐d_6_) δ: 21.4 (CH_3_), 52.8 (CH_2_), 96.9, 121.8, 123.1 (CH), 125.7 (CH), 127.7 (CH), 129.5 (CH), 132.0 (CH), 133.09, 139.94, 145.06, 152.71, 156.2 (CH), 170.09. HRMS‐ESI [(M+H)^+^]: m/z calculated for C_22_H_19_N_5_O_3_: 402.1561; found: 402.1559.

##### 4‐(4‐(4‐Methoxybenzoyl)piperazin‐1‐yl)‐6‐Nitroquinoline‐3‐Carbonitrile (8 c)

Yield 84 %. Mp 168–170 °C. ^1^H NMR (DMSO‐d_6_) δ: 3.80 (s, 3H, OCH_3_), 3.81 (br s, 8H, 4×CH_2_), 7.02 (d, 2H, J=8.7 Hz, H‐3’, H‐5’), 7.48 (d, 2H, 8.7 Hz, H‐2’, H‐6’), 8.15 (d, 1H, J=9.2 Hz, H‐8), 8.51 (dd, 1H, J=9.2, 2.5 Hz, H‐7), 8.91 (d, 1H, J=2.5 Hz, H‐5), 8.95 (s, 1H, H‐2). ^13^C NMR (DMSO‐d_6_) δ: 52.8 (CH_2_), 55.8 (CH_3_), 99.6, 114.2 (CH), 118.4, 121.9, 123.1 (CH), 125.7 (CH), 127.9, 129.7 (CH), 132.0 (CH), 145.1, 152.7, 156.2 (CH), 160.2, 160.9, 169.9. HRMS‐ESI [(M+H)^+^]: m/z calculated for C_22_H_19_N_5_O_4_: 418.1510; found: 418.1513.

##### 6‐Nitro‐4‐(4‐(4‐(Trifluoromethyl)benzoyl)piperazin‐1‐yl)Quinoline‐3‐Carbonitrile (8 d)

Yield 81 %. Mp 163–165 °C. ^1^H NMR (CDCl_3_) δ: 3.5–4.3 (m, 8H, 4×CH_2_), 7.6 (d, 2H, J=7.9 Hz, H‐3’, H‐5’), 7.7 (d, 2H, J=7.9 Hz, H‐2’, H‐6’), 8.2 (d, 1H, J=9.1 Hz, H‐8), 8.5 (dd, 1H, J=9.1, 2.5 Hz, H‐7), 8.9 (s, 1H, H‐2), 9.0 (d, 1H, J=2.5 Hz, H‐5). ^13^C NMR (CDCl_3_) δ: 52.9 (CH_2_), 98.3, 117.2, 121.3 (CH), 122.1, 125.4 (CH), 125.9 (m, J_C−F_=3.4 Hz, CH), 126.2 (m, J_C−F_=253 Hz), 127.6 (CH), 132.2 (m, J_C−F_=32 Hz), 132.6 (CH), 138.4, 145.5, 152.7, 155.4 (CH), 160.1, 169.3. HRMS‐ESI [(M+H)^+^]: m/z calculated for C_22_H_16_F_3_N_5_O_3_: 456.1278; found: 456.1278.

##### 4‐(4‐(4‐Chlorobenzoyl)piperazin‐1‐yl)‐6‐Nitroquinoline‐3‐Carbonitrile (8 e)

Yield 68 %. Mp 172–174 °C. ^1^H NMR (CDCl_3_) δ: 3.74–4.08 (br m, 8H, 4×CH_2_), 7.45 (s, 4H, H‐2’, H‐3’, H‐5’, H‐6’), 8.22 (d, 1H, J=9.2 Hz, H‐8), 8.53 (dd, 1H, J=9.2, 2.4 Hz, H‐7), 8.89 (s, 1H, H‐2), 8.96 (d, 1H, J=2.5 Hz, H‐5). ^13^C NMR (CDCl_3_) δ: 53.0 (CH_2_), 98.2, 117.3, 121.4 (CH), 122.1, 125.4 (CH), 128.8 (CH), 129.1 (CH), 132.6, 133.1, 136.6 (CH), 145.5, 152.7, 155.5 (CH), 160.1, 169.8. HRMS‐ESI [(M+H)^+^]: m/z calculated for C_21_H_16_ClN_5_O_3_: 422.1014; found: 422.1011.

##### 4‐(4‐(4‐Fluorobenzoyl)piperazin‐1‐yl)‐6‐Nitroquinoline‐3‐Carbonitrile (8 f)

Yield 80 %. Mp 174–176 °C. ^1^H NMR (CDCl_3_) δ: 3.72–4.08 (br m, 8H, 4×CH_2_), 7.07–7.21 (m, 2H, H‐2’, H‐6’), 7.47–7.57 (m, 2H, H‐3’, H‐5’), 8.22 (d, 1H, J=9.2 Hz, H‐8), 8.53 (dd, 1H, J=9.2, 2.4 Hz, H‐7), 8.89 (s, 1H, H‐2), 8.96 (d, 1H, J=2.5 Hz, H‐5). ^13^C NMR (CDCl_3_) δ: 53.0 (CH_2_), 98.1, 115.9 (d, J_C−F_=22.0 Hz, CH), 117.3, 121.4 (CH), 122.1, 125.4 (CH), 129.7 (d, J_C−F_=8.6 Hz, CH), 130.8 (d, J_C−F_=3.1 Hz), 132.5 (CH), 145.5, 152.7, 155.5 (CH), 160.2, 163.8 (d, J_C−F_=251.5 Hz), 169.9. HRMS‐ESI [(M+H)^+^]: m/z calculated for C_21_H_16_FN_5_O_3_: 406.1310; found: 406.1313.

##### 4‐(4‐(4‐Bromobenzoyl)piperazin‐1‐yl)‐6‐Nitroquinoline‐3‐Carbonitrile (8 g)

Yield 65 %. Mp 176–177 °C. ^1^H NMR (CDCl_3_) δ: 3.81 (s, 4H, 2×CH_2_), 3.98 (s, 4H, 2×CH_2_), 7.39 (d, 2H, J=8.4 Hz, H‐3’, H‐5’), 7.62 (d, 2H, J=8.4 Hz, H‐2’, H‐6’), 8.22 (d, 1H, J=9.1 Hz, H‐8), 8.53 (dd, 1H, J=9.2, 2.5 Hz, H‐7), 8.89 (s, 1H, H‐2), 8.96 (d, 1H, J=2.5 Hz, H‐5). ^13^C NMR (CDCl_3_) δ: 52.9 (CH_2_), 98.2, 117.2, 121.3 (CH), 122.1, 124.8, 125.4 (CH), 129.0 (CH), 132.1 (CH), 132.6 (CH), 133.6, 145.5, 152.7, 155.5 (CH), 160.1, 169.8. HRMS‐ESI [(M+H)^+^]: m/z calculated for C_21_H_16_BrN_5_O_3_: 466.0509; found: 466.0511.

##### 6‐Nitro‐4‐(4‐(3,4,5‐Trimethoxybenzoyl)piperazin‐1‐yl) quinoline‐3‐Carbonitrile (8 h)

Yield 94 %. Mp 183–185 °C. ^1^H NMR (CDCl_3_) δ: 3.81 (m, 4H, 2×CH_2_), 3.88 (s, 3H, OCH_3_), 3.91 (s, 6H, 2×OCH_3_), 4.01 (s, 4H, 2×CH_2_), 6.72 (s, 2H, H‐2’, H‐6’), 8.22 (d, 1H, J=9.2 Hz, H‐8), 8.53 (dd, 1H, J=9.2, 2.4 Hz, H‐7), 8.89 (s, 1H, H‐2), 8.97 (d, 1H, J=2.5 Hz, H‐5). ^13^C NMR (CDCl_3_) δ: 53.0 (CH_2_), 56.4 (CH_3_), 60.9 (CH_3_), 98.2, 104.6 (CH), 117.3, 121.4 (CH), 122.2, 125.3 (CH), 130.1, 132.6 (CH), 139.8, 145.5, 152.7, 153.6, 155.5 (CH), 160.2, 170.6. HRMS‐ESI [(M+H)^+^]: m/z calculated for C_24_H_23_N_5_O_6_: 478.1721; found: 478.1720.

##### 4‐(4‐(3‐Chloro‐4‐Fluorobenzoyl) piperazin‐1‐yl)‐6‐Nitroquinoline‐3‐Carbonitrile (8 i)

Yield 86 %. Mp 170–172 °C. ^1^H NMR (CDCl_3_) δ: 3.6–4.2 (br m, 8H, 4×CH_2_), 7.25 (t, 1H, J=8.5 Hz, H‐2’), 7.41 (ddd, 1 H, J=8.5, 4.5, 2.1 Hz, H‐6’), 7.60 (dd, 1H, J=6.9, 2.1 Hz, H‐5’), 8.23 (d, 1H, J=9.2 Hz, H‐8), 8.54 (dd, 1H, J=9.2, 2.5 Hz, H‐7), 8.90 (s, 1H, H‐2), 8.96 (d, 1 H, J=2.5 Hz, H‐5). ^13^C NMR (CDCl_3_) δ: 52.9 (CH_2_), 98.3, 117.1 (d, J_C−F_=21.6 Hz, CH), 117.2, 121.3 (CH), 122.0 (d, J_C−F_=17.9 Hz, C), 122.1, 125.4 (CH), 127.5 (d, J_C−F_=7.7 Hz, CH), 130.2 (CH), 131.8 (d, J=3.9 Hz, C), 132.6 (CH), 145.5, 152.7, 155.4 (CH), 159.2 (d, J_C−F_=254 Hz, C), 160.1, 168.5. HRMS‐ESI [(M+H)^+^]: m/z calculated for C_21_H_15_ClFN_5_O_3_: 440.0920; found: 440.0922.

##### 4‐(4‐(4‐(Dimethylamino)benzoyl)piperazin‐1‐yl)‐6‐Nitroquinoline‐3‐Carbonitrile (8 l)

Yield 50 %. Mp 173–175 °C. ^1^H NMR (CDCl_3_) δ: 3.03 (s, 6H, 2×CH_3_), 3.79–3.85 (m, 4H, 2×CH_2_), 3.98–4.05 (m, 4H, 2×CH_2_), 6.71 (d, 2H, J=8.8 Hz, H‐3’, H‐5’), 7.44 (d, 2H, J=8.8 Hz, H‐2’, H‐6’), 8.20 (d, 1H, J=9.2 Hz, H‐8), 8.52 (dd, 1H, J=9.2, 2.4 Hz, H‐7), 8.87 (s, 1H, H‐2), 8.97 (d, 1H, J=2.5 Hz, H‐5). ^13^C NMR (CDCl_3_) δ: 40.2 (CH_3_), 53.2 (CH_2_), 97.7, 111.3 (CH), 117.4, 121.1, 121.6 (CH), 122.1, 125.2 (CH), 129.6 (CH), 132.4 (CH), 145.3, 151.9, 152.7, 155.6 (CH), 160.2, 171.6. HRMS‐ESI [(M+H)^+^]: m/z calculated for C_23_H_22_N_6_O_3_: 431.1826; found: 431.1825.

### NCI60 Antiproliferative Screenings

#### Compound Selection Guidelines and Sample Preparation for Screening

The compounds chosen for screening were selected based on strict and specific criteria. Generally, submission is encouraged for molecules that introduced new structural features, such as novel heterocyclic ring systems and privileged scaffolds, to the NCI collection, as well as for those developed through computer‐aided drug design. When dealing with a series of analogues, preference is usually given to selecting the compound expected to yield the most significant information. Conversely, compounds with certain characteristics are discouraged from submission, including those with excessive flexibility, non‐drug‐like functional groups (such as nitroso, diazo, imine), and chemical components that could compromise the reliability of the assays (PAINS).^[61]^ All the submitted compounds were selected by NCI which, upon receipt a sample of 10 mg each, assigned the following identifying NSC codes: **13**, NSC847727; **8 a**, NSC847732; **8 b**, NSC847728; **8 c**, NSC847729; **8 d**, NSC847730; **8 e**, NSC847731; **8 f**, NSC847733; **8 g**, NSC847911; **8 h**, NSC84773; **8 i**, NSC847735; **8 l** (p‐DMA), NSC847734. Standard operating procedure for sample preparation, including sample concentration and volume requirements, solubilization and plating standard operation procedures, are detailed in.^[62]^

#### NCI‐60 Human Tumor Cell Lines Screen: One‐Dose Assay

All compounds submitted to NCI were first assayed in a one‐dose screen (concentration of 10^−5^ M) against the full NCI60 panel. As reported in detail in,^[26]^ the sixty cell lines tested are divided in nine subpanels each belonging to a different kind of cancer affecting humans: leukemia, non‐small cell lung cancer (NSCLC), colon, central nervous system (CNS), melanoma, ovarian, renal and breast cancers. This type of assay aims to determine the G % (growth inhibition percent) of the compounds against the targeted cells. The results were plotted in a one‐dose graph showing the G % of the single compound against the 60 cell lines. This first assay was considered passed only for the most promising compounds (satisfaction of predetermined threshold criteria); in this case, the compound passed to the five‐dose screen (for further experimental details about the standardized assay procedures, see ref.^[27]^).

### In Silico Studies

#### Ligand Preparation

LigPrep, implemented in the Maestro Suite by Schrödinger, is a robust and rapid collection of tools designed for preparing high‐quality small molecule ligand structures for structure‐based virtual screening and other computational workflows. It expands tautomeric and ionization states, ring conformations, and stereoisomers based on the input information to generate corresponding low‐energy 3D conformations of the molecule. For each ligand, all possible tautomers and stereoisomers were generated while retaining specified chiralities. Unwanted molecules were removed, as water or counter ions. Ionization states were computed for a pH range of 7.0±0.4 using the default Epik method, with ionization states maintained as specified in the input structure. Subsequently, the Optimized Potentials for Liquid Simulations (OPLS 2005) force field was employed to minimize the energy of the ligands.[^63^, ^64^] The output structures were then readily available for the virtual screening.

#### Protein Preparation

The X‐Ray crystal structures of the targets of interest investigated in this study were retrieved from the Protein Data Bank as .pdb files,[^32^, ^33^] with the following PDB codes: EGFR (PDB id: 1 M17);^[34]^ VEGFR‐2 (PDB id: 3WZD);^[35]^ RET (PDB id: 6NEC);^[36]^ c‐KIT (PDB id: 6GQK);^[37]^ c‐MET (PDB id: 3LQ8);^[38]^ AXL (PDB id: 7DXL);^[39]^ PDGFRA (PDB id: 6JOL);^[40]^ FGFR1 (PDB id: 4F63);^[41]^ PDL1 (PDB id: 5 N2D);^[42]^ ALK1 (PDB id: 3MY0);^[43]^ PI3Kα (PDB id: 7 K6 M);^[44]^ PI3 Kδ (PDB id: 4XE0);^[45]^ AKT/PKB (PDB id: 3OCB);^[46]^ mTOR (PDB id: 4JSX);^[47]^ B‐RAF (PDB id: 1UWH);^[48]^ C‐RAF (PDB id: 3OMV);^[49]^ BCL‐2 (PDB id: 6QGK);^[50]^ Bcl‐XL (PDB id: 3QKD);^[51]^ LSD1 (PDB id: 5LGN).^[52]^ The conventional .pdb structure file format is unsuitable for direct use in molecular modelling studies (presence of water, solvent and buffer molecules, metal ions, cofactors). For this reason, the proteins were prepared and refined using the Protein Preparation Wizard Task in the Schrödinger software with default settings.[^64^, ^65^, ^66^] In detail, this procedure involved several sequential steps. Each pdb structure was imported in Maestro and pre‐processed as follows (Preprocess Panel): bond orders, including the Het group, were assigned; all water molecules were deleted; protonation of the heteroatom states was carried out using the Epik‐tool (with the pH set at biologically relevant values, i. e., at 7.0±0.4); disulfide bonds were generated; missing loops and side chain near the binding site were generated using Prime. In the subsequent step (Analyze Panel), multimeric complexes were simplified removing redundant subunits. In the last step (Optimize and Minimize Panel), the H‐bond network was then optimized, and the structures were finally subjected to a restrained energy minimization step (RMSD of the atom displacement for terminating the minimization was 0.3 Å) using the OPLS 2005 force field.^[64]^

#### Structure‐Based Studies: Induced Fit Docking (IFD) Simulations

An Induced Fit Docking (IFD) simulation was performed on the top‐ranked targets identified during the VSW phase using Schrödinger's Induced Fit Docking tool, which is well‐regarded for its precision in accounting for the flexibility of both the ligand and the receptor. The validated IFD protocol from Schrödinger was applied to the receptor model, which had been refined beforehand using the Protein Preparation Wizard. The IFD score, calculated as IFD score=1.0×Glide Gscore+0.05×Prime Energy, which combines the protein‐ligand interaction energy with the total system energy, was computed and used to rank the IFD poses under default settings.[^67^, ^68^, ^69^, ^70^, ^71^] The docking procedure successfully re‐docked the original ligands into the receptor binding pockets with a root mean square deviation (RMSD) of less than 0.50 Å.

#### Structure‐Based Studies: XP Cross‐Docking Validation

To validate the binding specificity of the compounds, a cross‐docking analysis was carried out using the Glide docking protocol within the Maestro suite (Schrödinger, LLC). In the docking process, each ligand was docked against all 19 target proteins in extra precision (XP) mode (cross‐docking procedure). The docking was performed for all ligand‐target pairs, and the results were analysed to assess the docking scores and rank the inhibitors for each target.

#### Structure‐Based Studies: Molecular Dynamic Simulation

The molecular dynamics simulations were performed to investigate the effects of the solvent system of the stability of protein–ligand complex structure. These were performed using the explicit‐solvent molecular dynamics Desmond package provided by Schrödinger in Maestro version 13.8.155, MMshare Version 6.4.195, Release 2023–4,^[56]^ available for Platform Linux‐x8564. The simulations were conducted on a Dell Inc. Precision 7960 Tower equipped with an Intel®Xeon®w9‐3475X×72 processor and an NVIDIA corporation graphics processing unit running on Ubuntu 22.04.4 LTS 64 bit. Each thermodynamic chemical system simulated occupied a three‐dimensional volume of space of a specified size, with each atom represented by a particle at a specific position in that space. As starting positions for the complexes, the best‐docked poses of each ligand (**8 c**, **8 g**, and reference co‐crystallized ligands) in complex with the receptor of interest (AXL, C‐RAF, BCL‐2, LSD1, see previous sections for PDB codes) were retrieved from Induced Fit Docking studies. The System Builder panel was employed to set up each ligand‐protein complex. The predefined simple point charge (SPC) water model was selected as the solvent method to solvate and neutralize the system. The simulation box size was defined automatically by minimizing the volume of the box, selecting an orthorhombic shape, and using the Buffer method to optimize its size (the simulation box size was calculated with a given buffer distance of 10 Å×10 Å×10 Å between the solute structures and the simulation box boundary). The obtained solvated systems were loaded into the Molecular Dynamics tool panel. The runs were conducted for a simulation time of 100 ns using default OPLS4 force field, with an approximate maximum of 1000 frames in the trajectory. Default thermodynamic parameters were used, which provide a good balance between accuracy and performance: the constant‐temperature‐constant‐pressure (NPT) ensemble class was selected, setting the temperature to 300 K and the pressure to 1,013.25 bar. The systems were relaxed using the default protocol before running the simulation (automated minimization and equilibration). At the end of each simulation, the Trajectory Player was run to perform a visual inspection of the trajectory. Each output file was then analysed through the Simulation Quality Analysis panel to confirm the reliability of the experiment by computing the total and potential energy, the temperature, pressure and volume, which were confirmed to fluctuate around a constant value across the whole simulation (slope 0). Then, through the Simulation Interaction Diagram tool graphical representations of all computed parameters during the job were provided, including the following default geometric properties: Root Mean Square Deviation (RMSD) for both protein (Cα, backbone, side chains or heavy atoms) and ligands; Root Mean Square Fluctuation (RMSF) for protein residues and ligand; protein‐ligand contacts; ligand torsion.

#### ADME and Drug‐Likeness Parameters Prediction

QikProp tool is a quick, accurate, and easy‐to‐use program for predicting absorption, distribution, metabolism, and excretion (ADME) properties. QikProp predicts physically significant descriptors and pharmaceutically relevant properties of organic molecules, either individually or in batches and is available in Maestro suite – Schrödinger.^[20]^ Input structures as .sdf files were firstly prepared and optimized using the LigPrep task (see above section) and then processed with the QikProp tool in a normal mode. Selected ADME parameters, the bioavailability radar and BOILED‐Egg plots were obtained through SwissADME server (http://www.swissadme.ch) by uploading the SMILES string for each compound.

## Conclusions

In this study, we successfully designed, synthesized, and evaluated a novel series of 4‐(4‐benzoylpiperazin‐1‐yl)‐6‐nitroquinoline‐3‐carbonitrile compounds (**8 a**–**l**) as potential anticancer agents by a molecular hybridization approach.

A preliminary in silico ADMET and drug‐likeness assessments performed on the designed database displayed as the compounds favorable properties, prompting us to identify an appropriate synthetic strategy to afford them. The whole series of derivatives, isolated with adequate purity, was selected for the in vitro evaluations by the National Cancer Institute, which revealed potent anticancer activity, particularly against the renal cell carcinoma cell line UO‐31. In particular compounds **8 c** and **8 g** exhibited significant growth inhibition without excessive cytotoxicity. Interestingly, contrary to the rest of the series, **8 g** displayed potent anticancer activity also against other resistant and aggressive cancer types, such as NSCL, central nervous system and breast cancers (G % around 0 value).

The extensive induced fit molecular docking and dynamics studies provided insights into the mechanisms of action, highlighting strong interactions with key oncogenic proteins involved in RCC progression, as AXL, C‐RAF and LSD1. The detailed analysis of the ligand‐protein contacts revealed the most important structural portions involved in the complex's formation.

These promising results underscore the potential of these hybrid molecules as a foundation for developing new anticancer therapies. Further investigation, as in vitro assays on the specific targets as well as metabolic investigation on the NO_2_ group stability, will guide future lead‐optimization processes to enhance their therapeutic efficacy and selectivity.

## Conflict of Interests

The authors declare no conflict of interest.

## Supporting information

As a service to our authors and readers, this journal provides supporting information supplied by the authors. Such materials are peer reviewed and may be re‐organized for online delivery, but are not copy‐edited or typeset. Technical support issues arising from supporting information (other than missing files) should be addressed to the authors.

Supporting Information

## Data Availability

The data that support the findings of this study are available in the supplementary material of this article.
