# Supplementary material for: Design, Synthesis, and In Silico Insights of new 4‐Piperazinylquinolines as Antiproliferative Agents against NCI Renal Cancer Cell Lines
Source: ChemistryOpen. 2025 Feb 5;14(8):e202400518. doi: 10.1002/open.202400518 (PMC12368896; doi:10.1002/open.202400518)

ChemistryOpen

Supporting Information

Design, Synthesis, and In Silico Insights of new 4-Piperazinylquinolines as Antiproliferative Agents against NCI Renal Cancer Cell Lines

Gabriele La Monica, Alessia Bono, Federica Alamia, Annamaria Martorana,* and Antonino Lauria

Supporting Information

Design, synthesis, and in silico insights of new 4-piperazinylquinolines as antiproliferative agents against NCI renal cancer cell lines

Gabriele La Monica,[a] Alessia Bono,[a] Federica Alamia,[a] Annamaria Martorana,*[a] and Antonino Lauria[a]

[a] Dr. G. La Monica, A. Bono, F. Alamia, Prof. Dr. Annamaria Martorana, Prof. Antonino Lauria,
Department of Biological, Chemical and Pharmaceutical Sciences and Technologies (STEBICEF),
University of Palermo
Viale delle Scienze, Ed. 17, I-90128 Palermo, Italy
E-mail: annamaria.martorana@unipa.it

Contents

Sr. No.	Description	Page no.
1.	ADMET and drug-likeness parameters predictions	**.xlsx file**
2.	Antiproliferative Activity Data (G%) for compounds **8a-l** against NCI60 cells	**2**
3.	In silico structure-based studies: Induced Fit Docking (IFD) and Molecular Dynamic simulations (additional figures and table)	**5**
4.	^{1}H and ^{13}C NMR spectra for compounds **13** and **8a-l**	**9**

Antiproliferative Activity Data for compounds 8a-l against NCI60 cells

Table SI-1. Growth inhibition percentage values (G%) provided by the National Cancer Institute for compounds **13** and **8a-l** tested against the full NCI60 panel. The red values referred to G% under the threshold value of 55%.

PANEL	CELL LINE	COMPOUNDS CODES/ NSC NUMBERS										
		13	8a	8b	8c	8d	8e	8f	8g	8h	8i	8l
		847727	847732	847728	847729	847730	847731	847733	847911	847736	847735	847734
Leukemia	CCRF-CEM	92,31	88,83	96,62	105,05	87,16	77,84	91,22	55,69	100,77	89,51	95,91
	HL-60(TB)	89,38	96,37	132,11	125,98	158,75	150,28	88,97	68,42	89,51	106,22	84,27
	K-562	89,54	92,08	100,81	99,14	94,13	85,75	86,23	**32,49**	96,36	78,64	86,54
	MOLT-4	114,04	78,33	111,37	134	95,25	124,08	74,07	**32,88**	86,02	75,38	85,58
	RPMI-8226	93,1	96,68	103,05	97,95	90,95	96,41	86,96	57,59	83,63	86,83	89,79
	SR	80,88	69,57	95,25	95,38	86,12	78,87	61,13	**40,76**	74,7	65,87	68,74
NSCLC	A549/ATCC	96,38	91,44	91,59	97,9	87,15	82,75	92,11	55,01	89,66	86,37	89,78
	EKVX	93,87	99,42	102,51	96,16	88,94	87,27	96,66	56,55	81,72	93,72	98,15
	HOP-62	106,69	84,4	102,78	97,7	97,71	97,94	84,8	**41,26**	109,9	102,16	92,15
	HOP-92	102,6	73,65	107,63	105,52	99,35	94,83	77,03	**-3,39**	70,89	88,52	83,34
	NCI-H226	69,67	106,9	81,45	82,17	72,02	68,64	92,91	**36,22**	106,26	97,19	103,86
	NCI-H23	93,16	100,34	100,28	96,76	90,87	91,29	91,53	68,55	88,14	92,6	94,4
	NCI-H322M	113,54	125,88	103,53	104,07	103,03	95,35	119,09	74,73	111,64	117,98	122,88
	NCI-H460	97,18	103,59	101,67	103,55	95,13	97,1	102,82	**40,65**	101,09	101,97	98,78
	NCI-H522	91,77	85,43	91,99	92,22	75,68	76,46	79,54	**26,26**	88,82	80,12	85,82
COLON CANCER	COLO-205	91,06	113,66	95,33	94,67	98,2	93,93	109,77	63,59	102,44	91,48	111,07
	HCC-2998	92,21	111,52	106,03	94,94	101,24	95,11	109,86	62,3	108,28	100,21	101,6
	HCT-116	95,49	104,79	106,98	115,3	94,36	97,72	108,44	**48,7**	107,58	94,5	111,16
	HCT-15	98,79	98,9	99,4	99,17	96,22	97,57	99,34	85,03	98,76	97,71	98,93
	HT29	90,87	93,29	89,28	96,97	98,8	90,81	90,71	63,53	101,3	86,38	90,09
	KM12	97,18	98,77	99,41	99,23	97,53	96,73	97,75	72,32	98,41	96,17	97,15
	SW-620	88,46	96,42	98,42	98,78	96,97	90	94,9	63,66	90,13	97,78	95,88
CNS CANCER	SF-268	97,21	99,13	108,27	99,36	91,46	96,36	108,82	**49,67**	81,94	101,72	104,11
	SF-295	91,96	89,48	88,9	86,55	76,48	74,53	91,02	**42,6**	95,59	87,18	90,7
	SF-539	91,77	92,41	99,49	97,81	93,71	86,96	88,25	**47,69**	78,93	86,5	86,31
	SNB-19	87,41	99,25	99,07	95,6	97,96	91,95	91,22	**54,69**	93,17	92,28	90,51
	SNB-75	96,05	111,78	109,44	95,12	101,65	96,05	128,92	**4,43**	97,78	132,31	141,99
	U251	85,49	94,44	100,11	94,92	91,63	91,91	91,11	**47,86**	91	90,46	89,09
MELANOMA	LOX_IMVI	82,78	91,79	97,55	94,79	82,61	84,61	90,75	67,3	85,89	88,8	93,71
	MALME-3M	88,85	105,94	106,79	94,73	96,83	93,76	97,8	56,66	99,47	97,2	102,12

	**M14**	101,08	109,19	108,05	105,27	99,21	97,73	95,48	73,67	103,05	101,13	93,39
	**MDA-MB-435**	99,71	101,29	99,11	98,91	97,25	96,44	101,86	75,49	101,02	101,74	99,59
	**SK-MEL-2**	105,41	100,16	110,34	107,26	105,55	98,08	99,82	**54,57**	108,78	104,04	103,44
	**SK-MEL-28**	108,16	111,61	100,51	102,32	94,93	95,77	100,88	74,49	113,08	108,29	102,09
	**SK-MEL-5**	83,11	80,65	86,14	91,38	67,87	71,07	80,58	**45,79**	75,22	72,44	81,87
	**UACC-257**	93,24	101,53	105,66	96,39	102,37	101,55	96,04	86,19	91,83	98,9	99,94
	**UACC-62**	87,6	93,4	82,34	91,04	72,29	77,38	89,1	54,99	91,92	79,45	88,78
**OVARIAN CANCER**	**IGROV1**	109,53	120,74	100,95	106,8	89,21	93,38	115,71	65,29	109,25	99,53	110,78
	**OVCAR-3**	105,84	112,14	119,38	109,11	113,65	112,56	117,48	85,4	104,37	117,66	118,88
	**OVCAR-4**	86,8	104,92	86,36	86,82	74,46	72,69	91,53	**41,22**	86,1	82,54	96,08
	**OVCAR-5**	100,9	101,92	97,65	97,27	94,46	92,46	102,12	74,98	103,15	104,3	106,11
	**OVCAR-8**	79,06	100,2	92,48	95,27	89,52	82,61	94,79	61,72	94,21	91,34	96,05
	**NCI/ADR-RES**	92,96	94,85	91,66	92,19	90,33	82,77	89,62	58,96	88,18	93,16	96,31
	**SK-OV-3**	150,08	116,83	117,2	89,96	98,85	95,03	126,18	**54**	110,14	130,77	139,24
**RENAL CANCER**	**786-0**	98,83	95,88	98,25	93,8	95,31	89,34	101,61	**47,95**	95,88	91,13	106,09
	**A498**	124,45	124,61	119,38	105,35	113,46	106,03	130,53	**25,97**	79,33	123,83	116,24
	**ACHN**	80,15	88,82	85,53	89,57	79,75	78,76	83,4	55,02	80,25	80,32	88,46
	**CAKI-1**	84,58	81,04	86,01	89,66	75,96	79,51	82,42	**31,87**	61,04	66,4	73,7
	**RXF-393**	93,59	94,56	92,85	95,7	83,15	76,24	92,35	**32,82**	77,33	83,69	92,39
	**SN12C**	88,11	89,73	90	88,24	88,88	84,17	92,85	58,7	88,69	90,62	88,9
	**TK-10**	102,11	102,01	102,82	97,09	110,8	101,11	97,81	63,76	92,27	94,48	97,06
	**UO-31**	62,52	69,49	**-70,71**	**-6,68**	61,18	**-69,88**	**-54,57**	**-19,4**	**-89,02**	63,37	**-46,38**
**PROSTATE CANCER**	**PC-3**	87,88	92,7	92,22	95,07	83,33	84,18	95,48	57,42	86,97	90,08	92,81
	**DU-145**	106,84	100,47	99,91	100,34	91,78	92,88	100,53	59,22	103,27	96,4	103,8
**BREAST CANCER**	**MCF7**	80,03	85,97	86,87	86,34	84,11	77,28	80,06	55,93	82,5	85,07	79,1
	**MDA-MB-231/ATCC**	92,38	85,65	89,4	93,36	79,13	82,1	79,29	**42,43**	74,86	83,59	81
	**HS-578T**	104,3	106,1	117,66	106,96	105,7	111,73	103,34	**7,71**	86,66	111,37	101,26
	**BT-549**	113,87	101,97	109,95	113,16	94,22	97,3	110,09	**39,87**	106,22	98,53	112,74
	**T-47D**	86,45	85,08	88,78	87,38	72,13	87,19	81,61	**35,06**	92,78	91,67	72,08
	**MDA-MB-468**	83,01	98,66	85,82	93,19	66,96	73,09	109,86	**35,96**	95,48	96,32	111,52
**Mean**		94,87	97,44	96,66	96,5	91,89	88,39	93,53	50,85	90,08	93,93	94,8
**Delta**		32,35	27,95	167,37	103,18	30,71	158,27	148,1	70,25	179,1	30,56	141,18
**Range**		87,56	56,39	202,82	140,68	97,57	220,16	185,1	105,59	202,1	68,94	188,37

Supporting Figure SI-2. One-dose mean graph (G%) of compound **8g** against the full NCI60 panel.

Developmental Therapeutics Program	NSC: D-847911 / 1	Conc: 1.00E-5 Molar	Test Date: Aug 21, 2023
One Dose Mean Graph	Experiment ID: 2308OS65		Report Date: Oct 11, 2023

Panel/Cell Line	Growth Percent
**Leukemia**	
CCRF-CEM	55.69
HL-60(TB)	68.42
K-562	32.49
MOLT-4	32.88
RPMI-8226	57.59
SR	40.76
**Non-Small Cell Lung Cancer**	
A549/ATCC	55.01
EKVX	56.55
HOP-62	41.26
HOP-92	-3.39
NCI-H226	36.22
NCI-H23	68.55
NCI-H322M	74.73
NCI-H460	40.65
NCI-H522	26.26
**Colon Cancer**	
COLO 205	63.59
HCC-2998	62.30
HCT-116	48.70
HCT-15	85.03
HT29	63.53
KM12	72.32
SW-620	63.66
**CNS Cancer**	
SF-268	49.67
SF-295	42.60
SF-539	47.69
SNB-19	54.69
SNB-75	4.43
U251	47.86
**Melanoma**	
LOX IMVI	67.30
MALME-3M	56.66
M14	73.67
MDA-MB-435	75.49
SK-MEL-2	54.57
SK-MEL-28	74.49
SK-MEL-5	45.79
UACC-257	86.19
UACC-62	54.99
**Ovarian Cancer**	
IGROV1	65.29
OVCAR-3	85.40
OVCAR-4	41.22
OVCAR-5	74.98
OVCAR-8	61.72
NCI/ADR-RES	58.96
SK-OV-3	54.00
**Renal Cancer**	
786-0	47.95
A498	25.97
ACHN	55.02
CAKI-1	31.87
RXF 393	32.82
SN12C	58.70
TK-10	63.76
UO-31	-19.40
**Prostate Cancer**	
PC-3	57.42
DU-145	59.22
**Breast Cancer**	
MCF7	55.93
MDA-MB-231/ATCC	42.43
HS 578T	7.71
BT-549	39.87
T-47D	35.06
MDA-MB-468	35.96
Mean	50.85
Delta	70.25
Range	105.59

Mean Growth Percent - Growth Percent

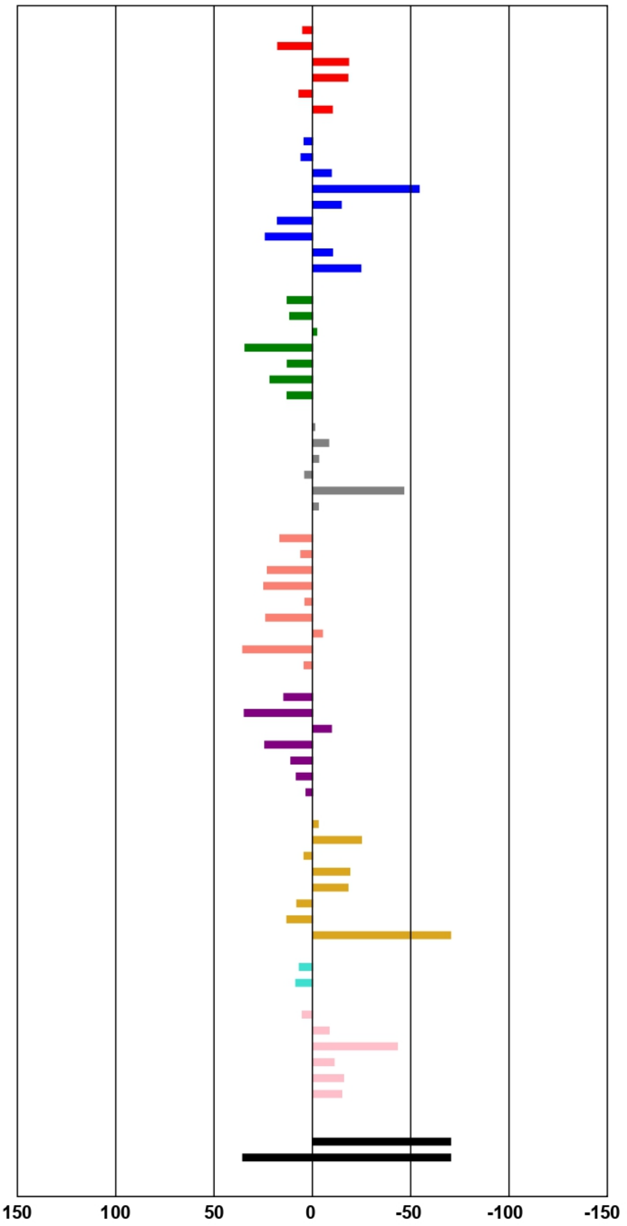

In silico structure-based studies: Induced Fit Docking (IFD) and Molecular Dynamic (MD) simulation (additional figures and tables)

Supporting Table SI-3. Full Induced Fit Docking results, including IFD, Glide and Prime Energy scores obtained for compounds **8c** and **8g** against the 19 investigated targets involved in RCC. For each target, the results related to the corresponding co-crystallized inhibitor are reported as control. In red the 4 best performing targets considered for MD studies are highlighted.

Target	Compound	IFD Score	Glide score	Prime Energy
EGFR (PDB id: 1M17)	CID176870	-665.26	-9.641	-13112.33
	8c	-654.55	-8.330	-12924.32
	8g	-654.46	-7.775	-12933.61
VEGFR-2 (PDB id: 3WZD)	CID9823820	-611.44	-12.577	-11977.28
	8c	-602.79	-9.619	-11863.34
	8g	-602.38	-9.357	-11860.52
RET (PDB id: 6NEC)	CID135423438	-665.93	-12.288	-13072.83
	8c	-662.14	-9.659	-13049.58
	8g	-661.08	-9.443	-13032.74
c-KIT (PDB id: 6GQK)	CID134814267	-673.74	-12.264	-13229.57
	8c	-663.92	-10.974	-13058.85
	8g	-663.09	-10.004	-13061.66
c-MET (PDB id: 3LQ8)	CID42642645	-606.71	-15.547	-11812.45
	8c	-594.76	-11.340	-11668.33
	8g	-592.92	-10.563	-11647.09
AXL (PDB id: 7DXL)	8c	-573.63	-11.152	-11249.52
	CID156613395	-571.11	-11.005	-11202.06
	8g	-568.87	-9.674	-11184.01
PDGFRA (PDB id: 6JOL)	CID5291	-610.41	-12.728	-11953.55
	8c	-600.27	-11.159	-11782.18
	8g	-600.00	-11.510	-11769.82
FGFR1 (PDB id: 4F63)	CID56973548	-626.23	-9.767	-12329.09
	8c	-615.05	-8.677	-12127.53
	8g	-612.94	-8.313	-12092.44
PDL1 (PDB id: 5N2D)	CID117941658	-567.20	-12.435	-11094.85
	8g	-562.29	-11.159	-11022.55
	8c	-560.26	-9.716	-11010.89
ALK1 (PDB id: 3MY0)	CID25195294	-659.74	-10.383	-12987.14
	8c	-657.58	-9.990	-12951.76
	8g	-654.96	-9.863	-12901.99

PI3Kα (PDB id: 7K6M)	**CID124193915**	-2010.69	-11.850	-39976.87
	**8g**	-1996.13	-10.239	-39717.73
	**8c**	-1996.10	-10.266	-39716.78
PI3Kδ (PDB id: 4XE0)	**CID11625818**	-1763.04	-10.849	-35043.80
	**8g**	-1760.70	-9.721	-35019.63
	**8c**	-1760.24	-9.252	-35019.81
AKT/PKB (PDB id: 3OCB)	**CID46870040**	-736.79	-10.537	-14525.11
	**8g**	-731.66	-8.168	-14469.89
	**8c**	-731.49	-8.934	-14451.09
mTOR (PDB id: 4JSX)	**CID51358113**	-2332.93	-13.080	-46395.40
	**8c**	-2327.08	-9.535	-46350.92
	**8g**	-2325.47	-8.337	-46342.64
B-RAF (PDB id: 1UWH)	**CID216239**	-580.15	-12.959	-11343.50
	**8g**	-572.35	-11.355	-11219.93
	**8c**	-572.16	-10.723	-11228.73
C-RAF (PDB id: 3OMV)	**CID11653652**	-551.35	-11.075	-10805.54
	**8c**	-551.26	-12.243	-10780.43
	**8g**	-549.25	-11.329	-10758.40
BCL-2 (PDB id: 6QGK)	**CID138393395**	-293.35	-7.977	-5707.50
	**8g**	-291.76	-8.392	-5667.29
	**8c**	-290.32	-7.620	-5654.03
BCL-XL (PDB id: 3QKD)	**CID24798804**	-350.00	-12.553	-6738.54
	**8c**	-335.07	-8.796	-6525.47
	**8g**	-333.86	-8.154	-6514.15
LSD1 (PDB id: 5LGN)	**8c**	-1444.67	-8.839	-28716.54
	**CID51049245**	-1443.16	-8.460	-28694.01
	**8g**	-1442.86	-9.124	-28674.74

Supporting Figure SI-4. 2D protein-ligand interaction diagrams obtained from Induced Fit Docking Studies (IFD) for **8c** and **8g** in complex with the best ranked targets AXL, C-RAF, BCL-2 and LSD1. The reference compounds are included for comparison.

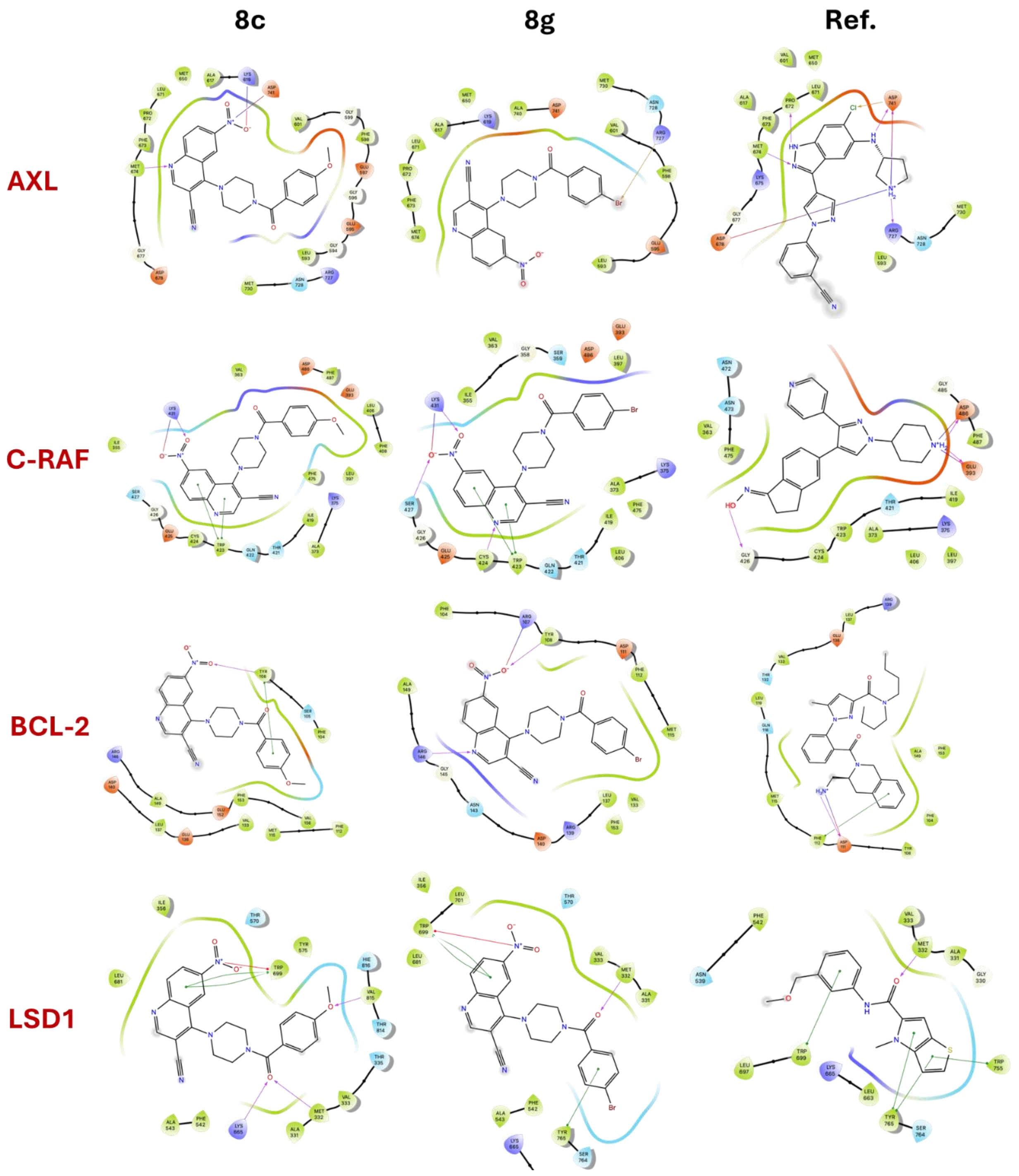

Supporting Figure SI-5. RMSF plots referred to the MD simulations of the control ligands (CID156613395 for AXL, CID11653652 for C-RAF, CID51049245 for LSD1) in complex with the corresponding target.

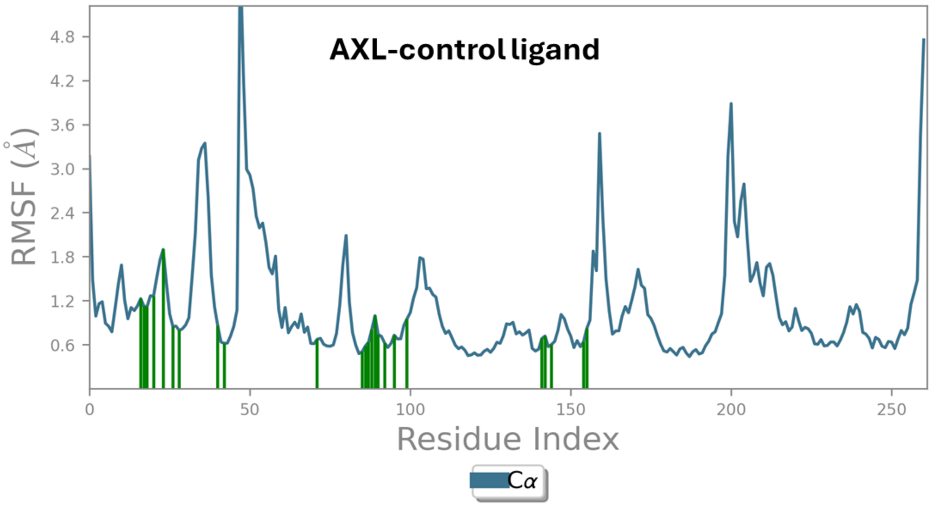

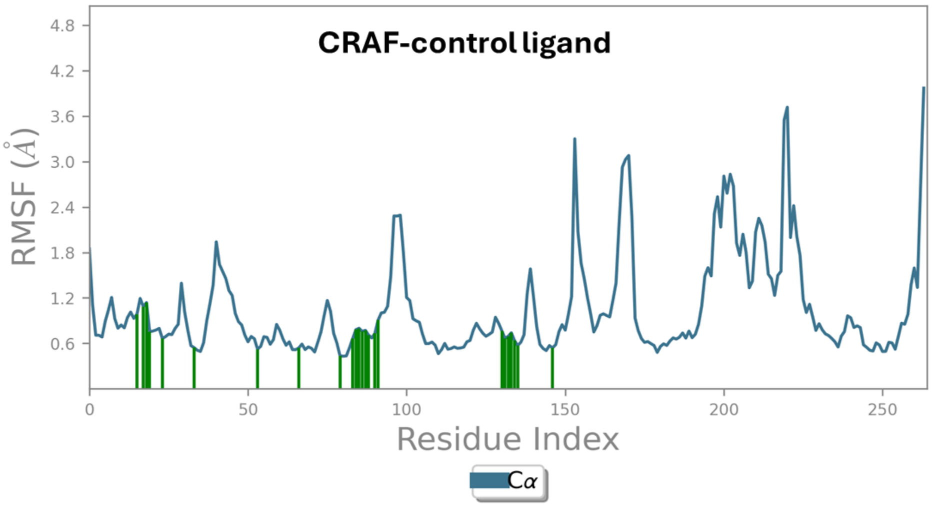

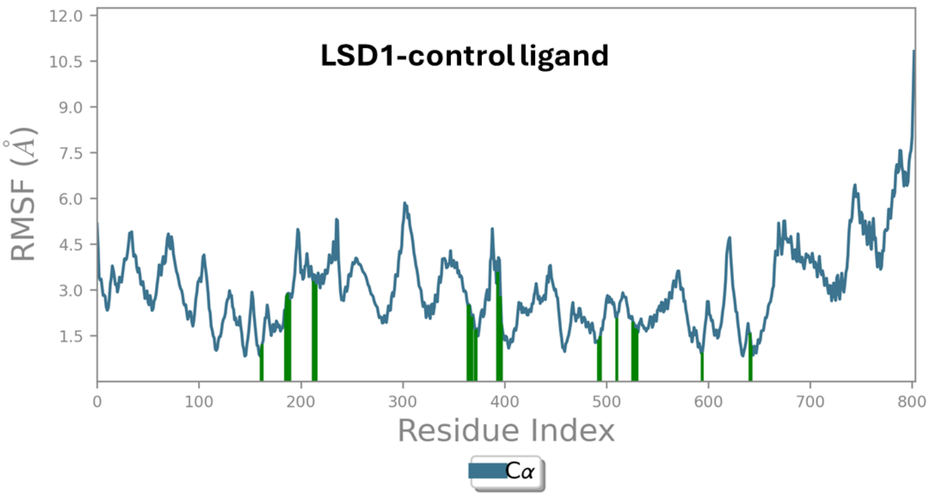

^{1}H and ^{13}C NMR spectra for compounds 13 and 8a-l

^{1}HNMR compound **13** ($CDCl_3$)

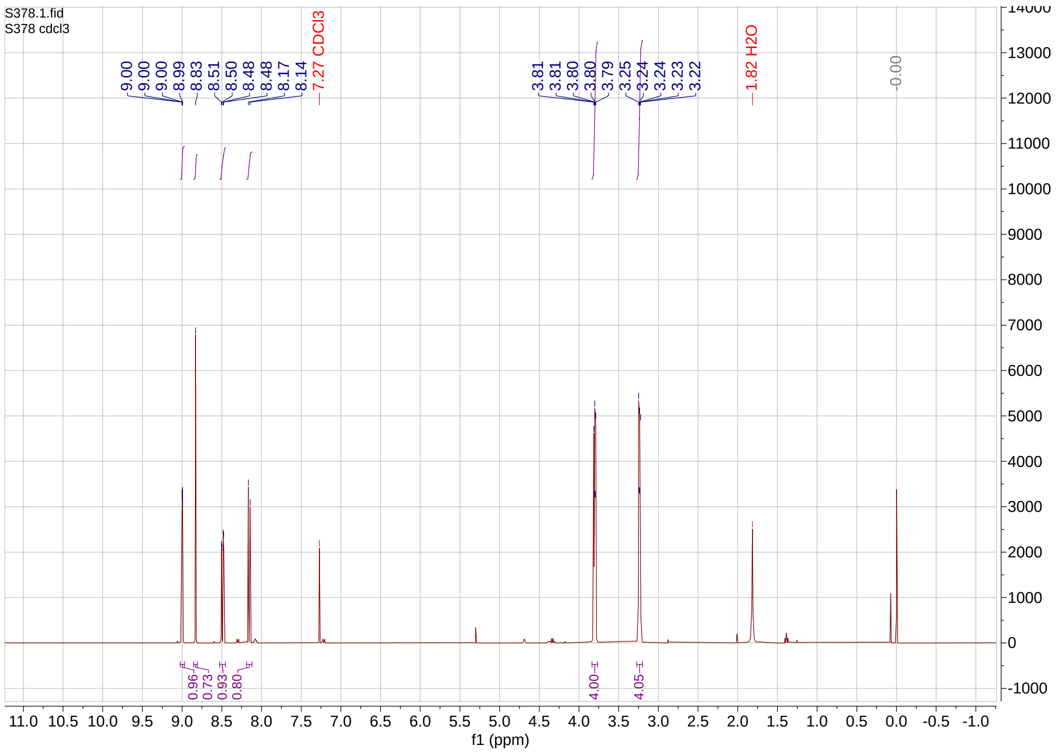

^{13}CNMR compound **13** ($CDCl_3$)

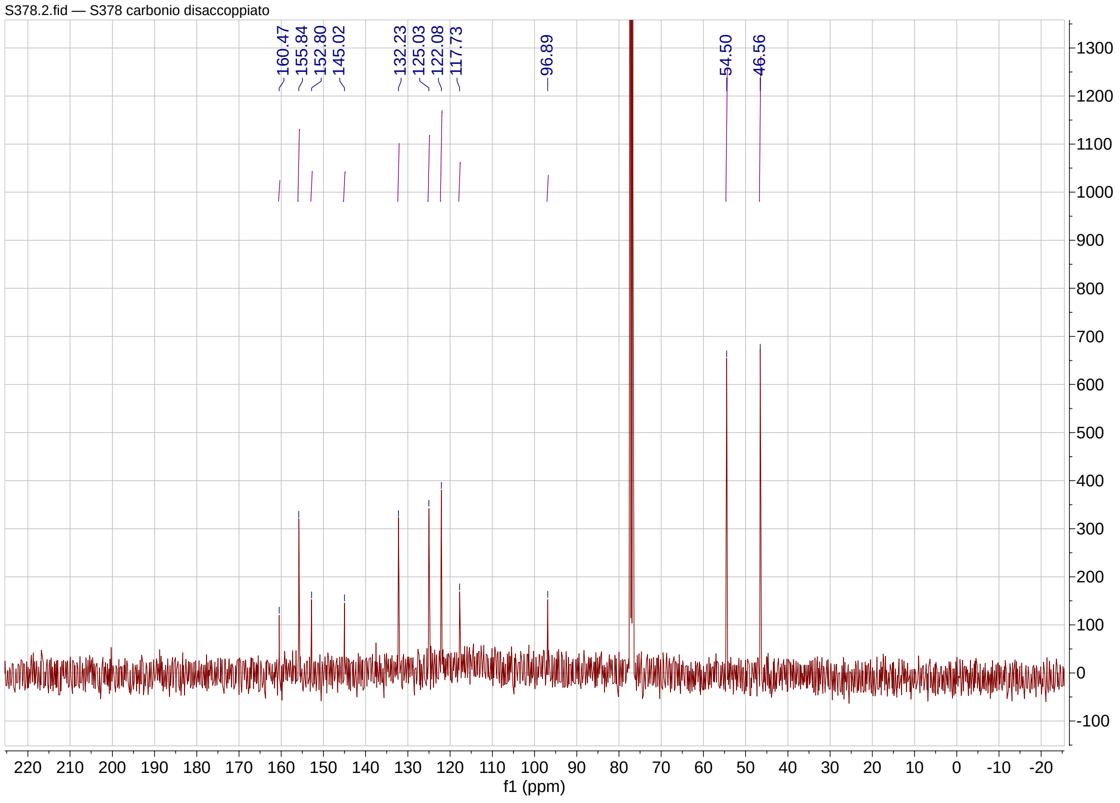

^{1}HNMR compound **8a** ($CDCl_3$)

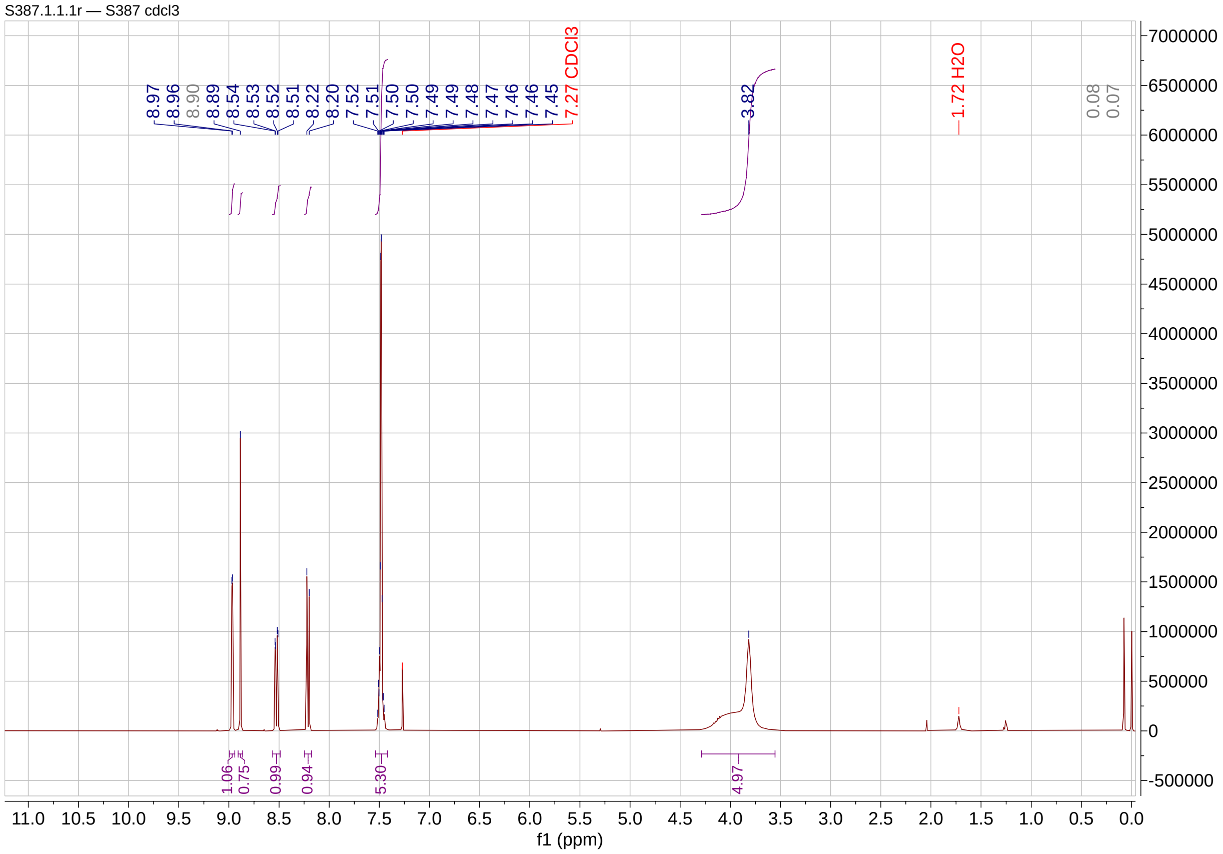

^{13}CNMR compound **8a** ($CDCl_3$)

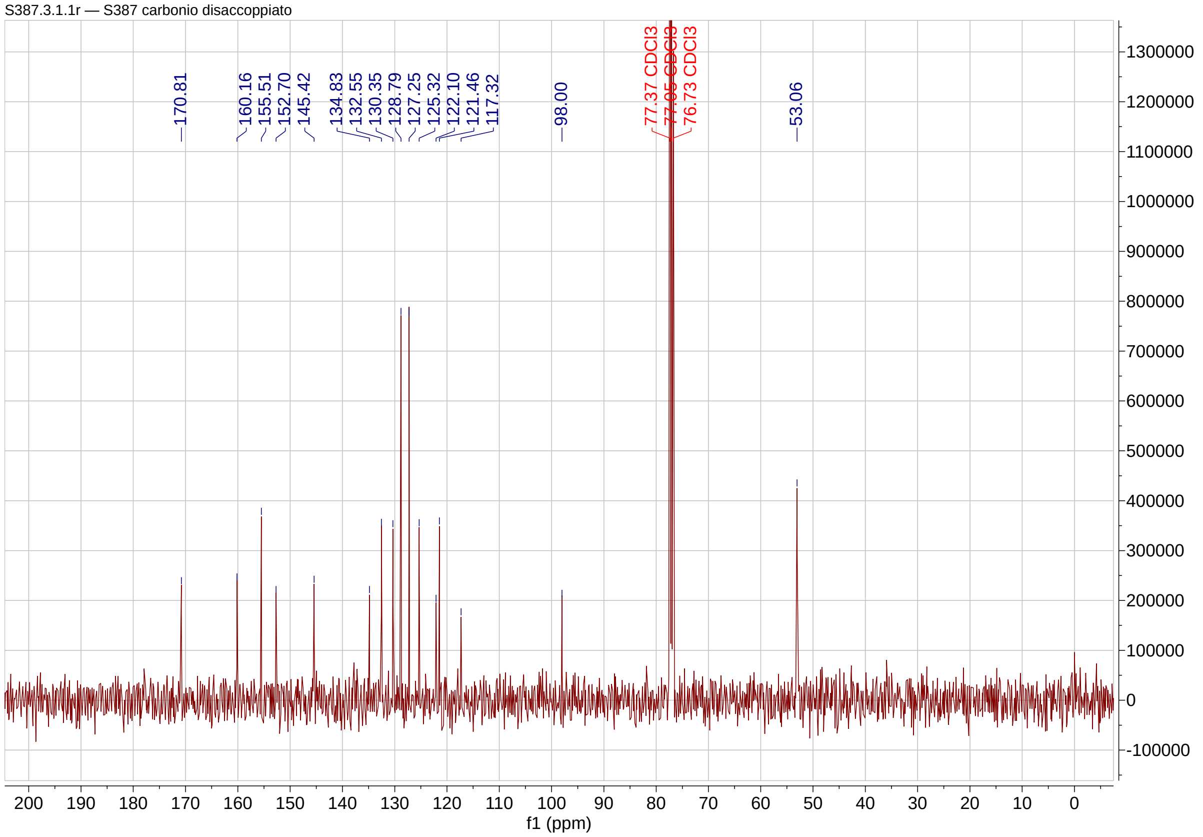

^{1}HNMR compound **8b** (DMSO-d_6)

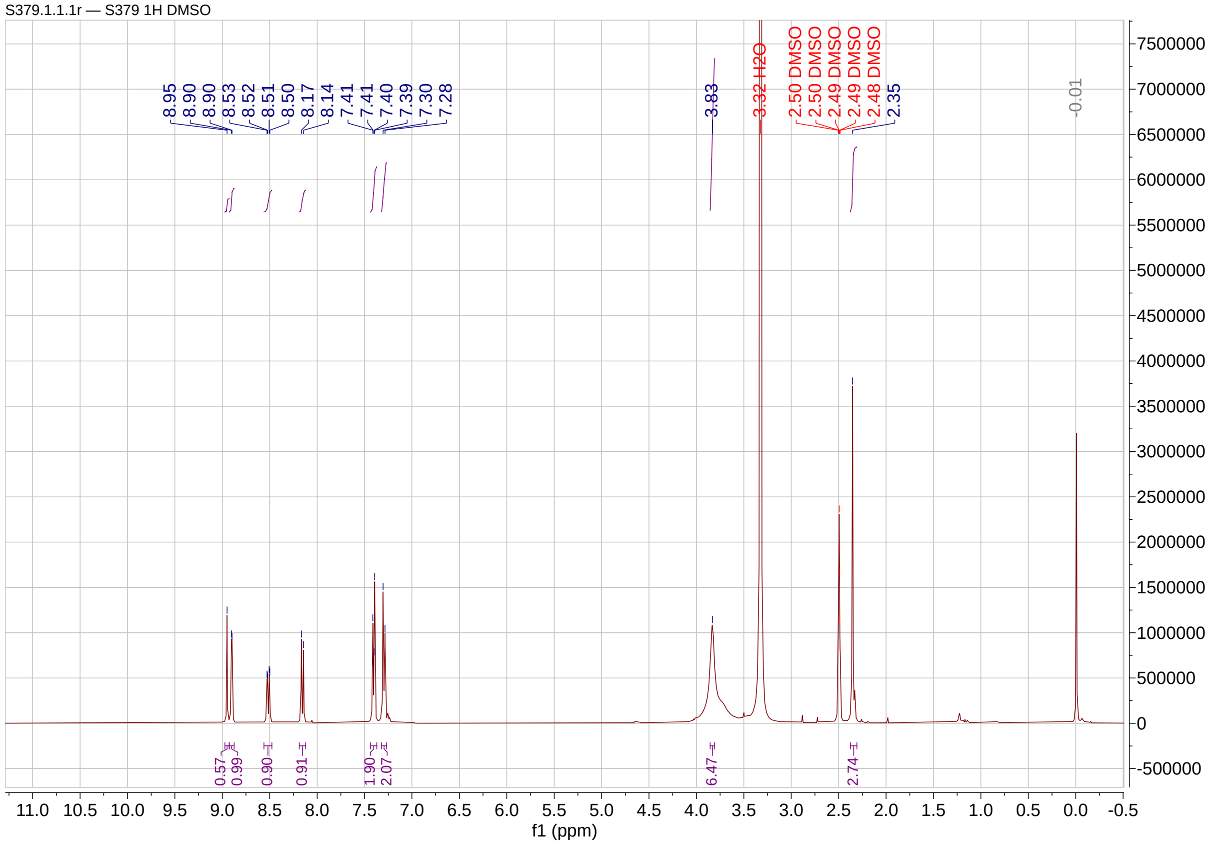

^{13}CNMR compound **8b** (DMSO-d_6)

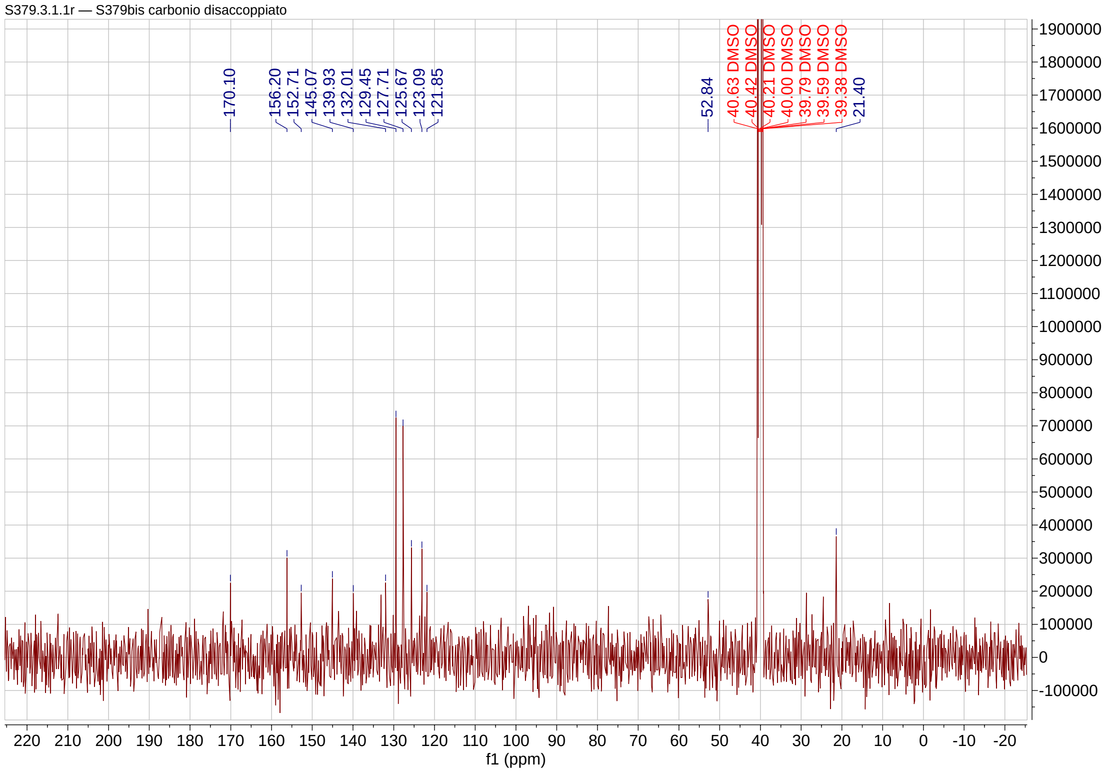

^{1}HNMR compound **8c** (DMSO-d6)

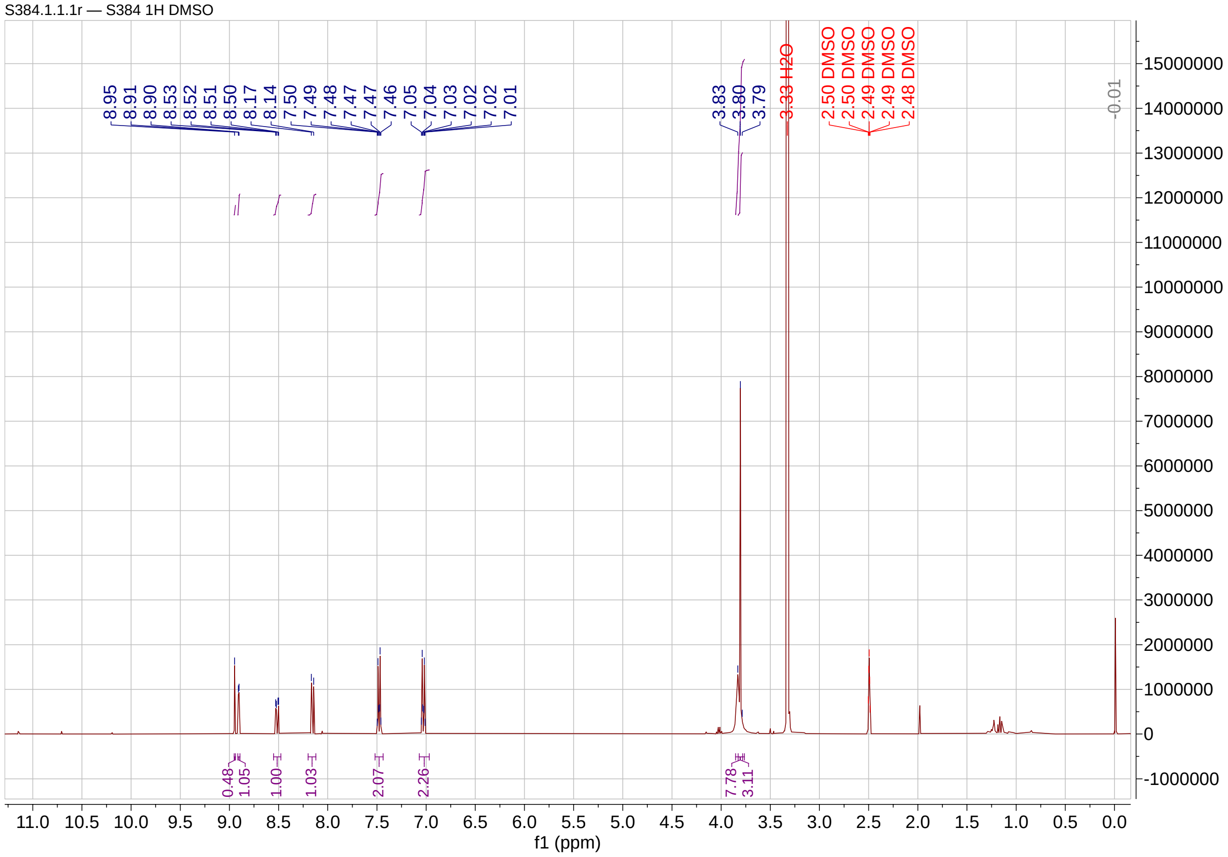

^{13}CNMR compound **8c** (DMSO-d_6)

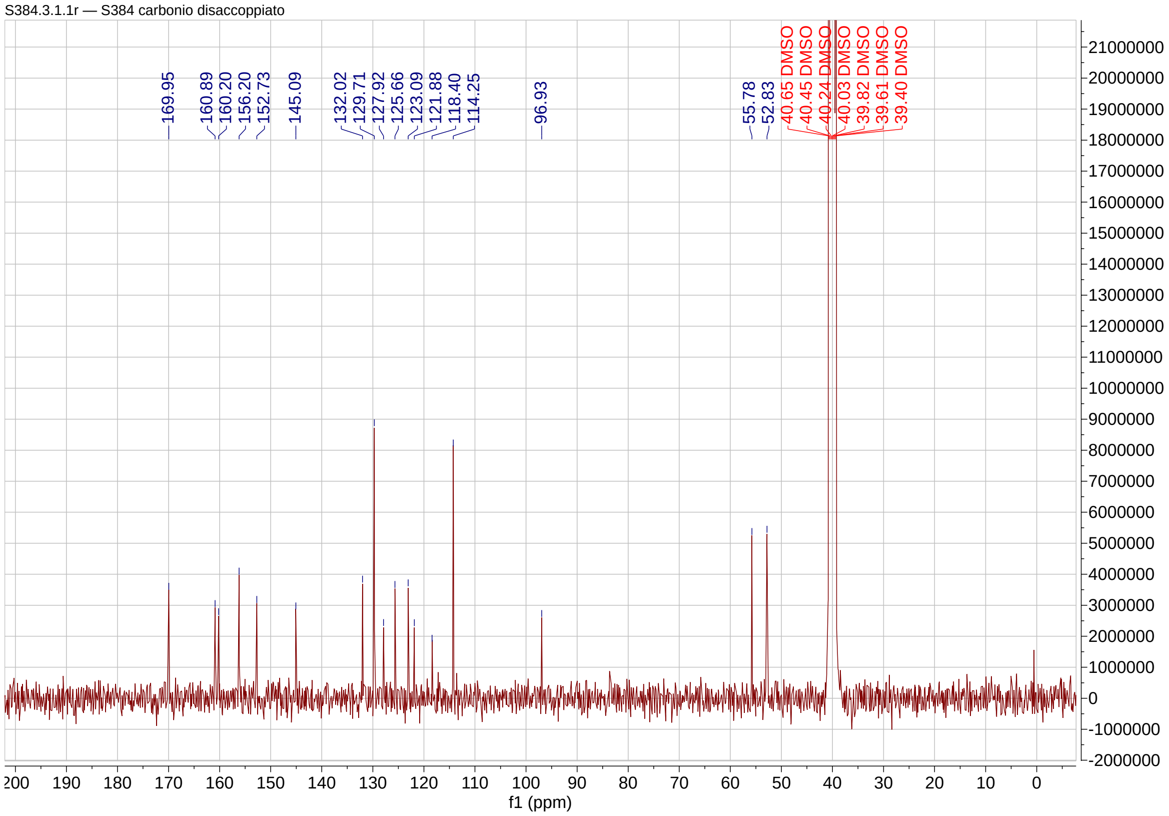

^{1}HNMR compound **8d** ($CDCl_3$)

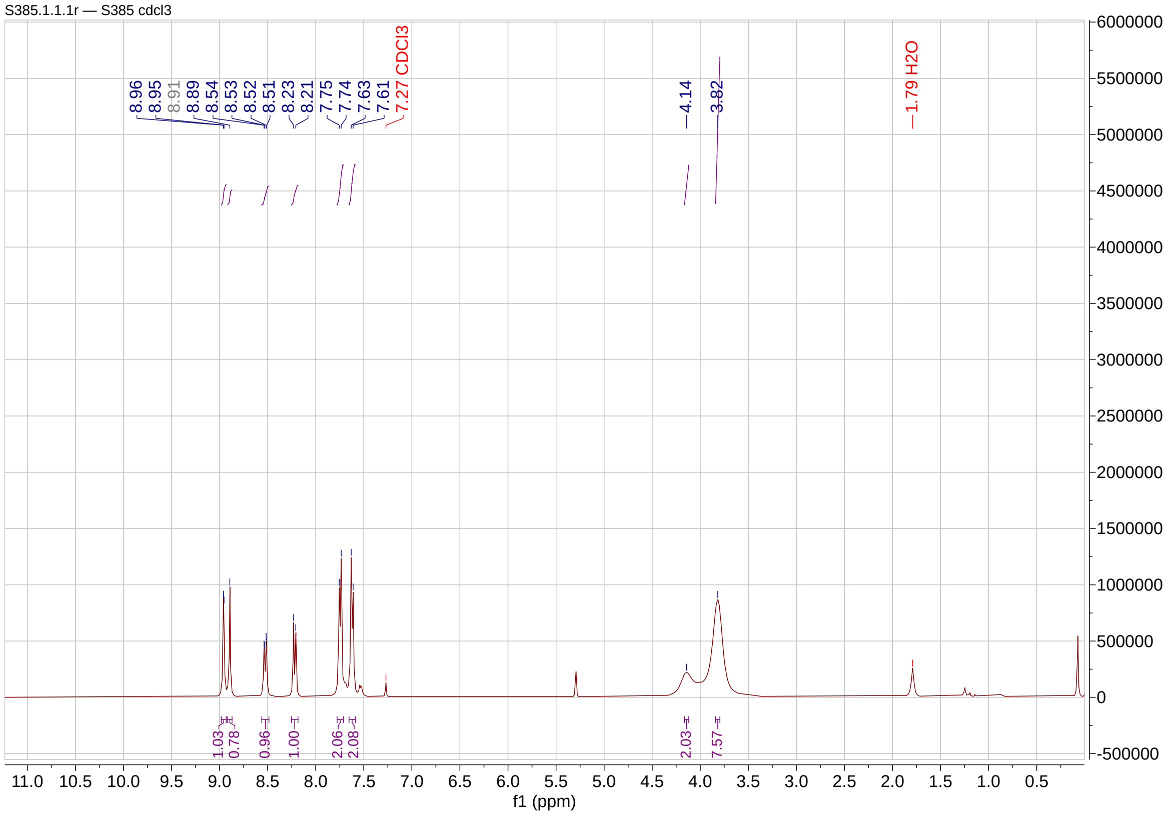

^{13}CNMR compound **8d** ($CDCl_3$)

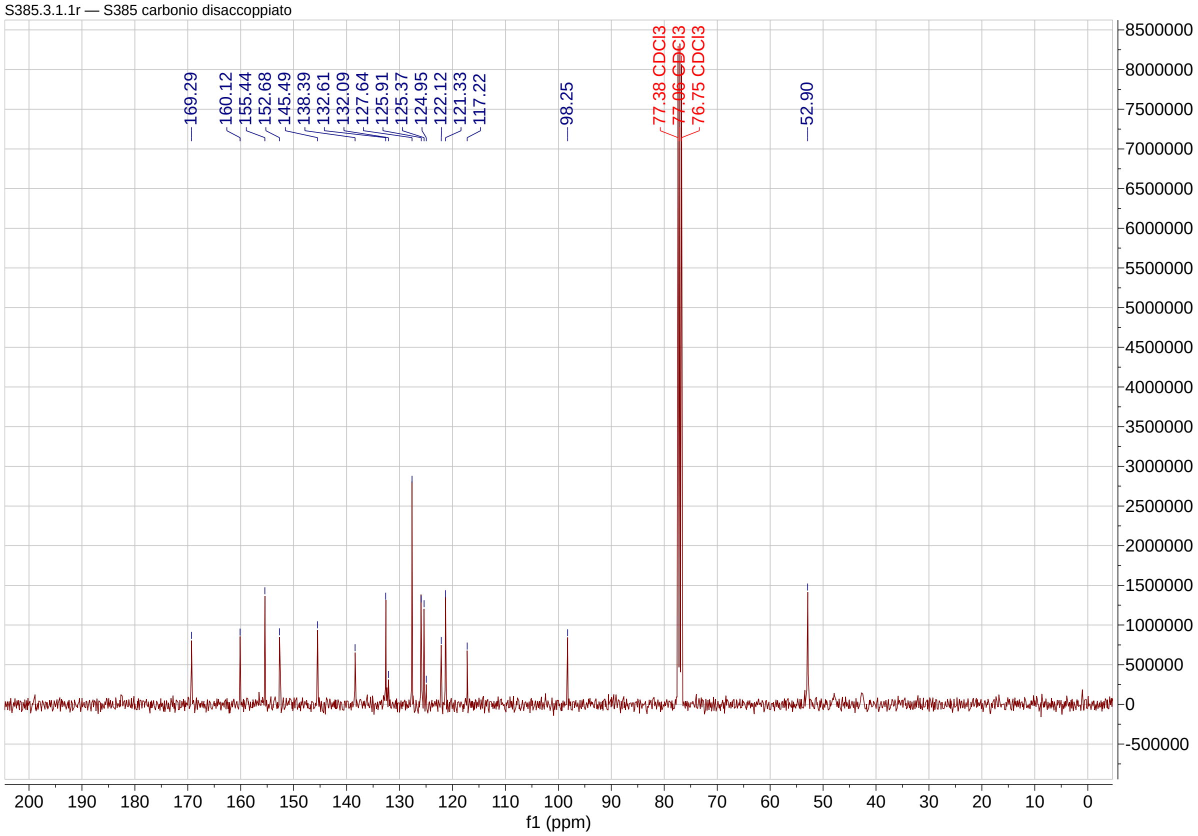

^{1}HNMR compound **8e** ($CDCl_3$)

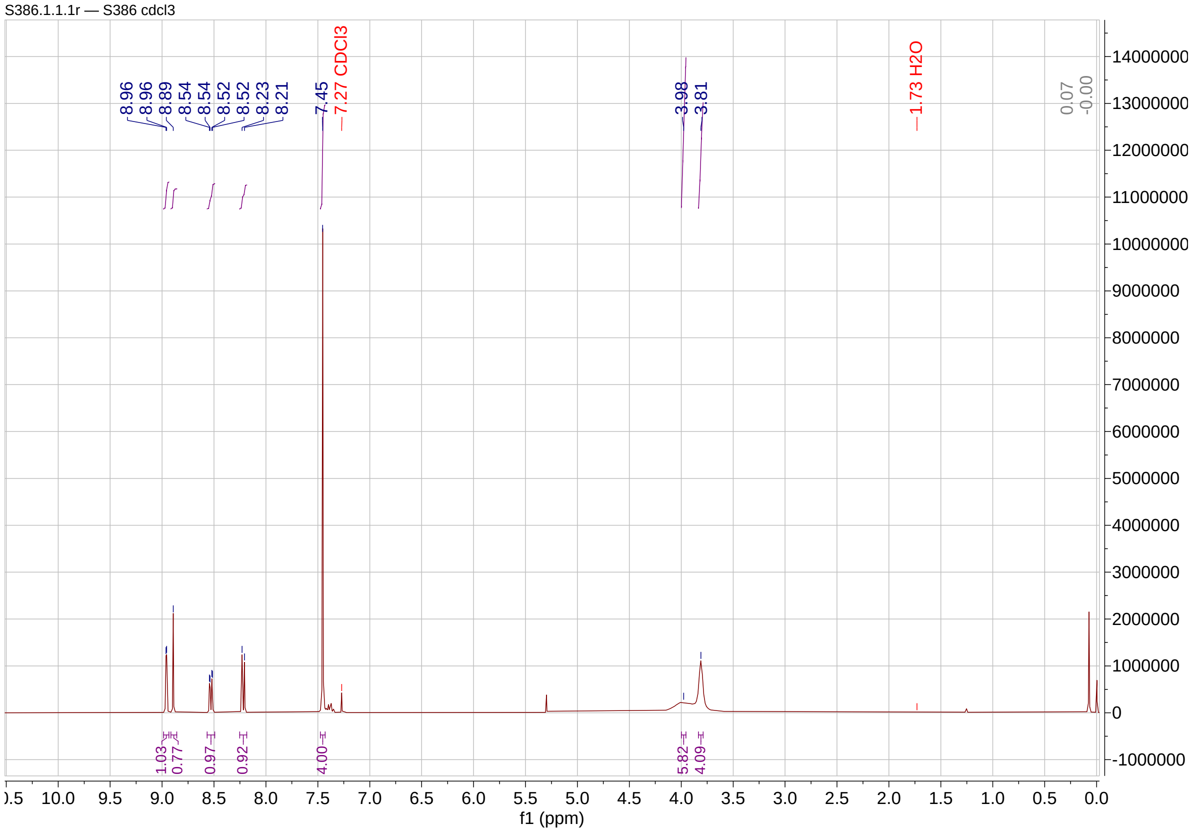

^{13}CNMR compound **8e** ($CDCl_3$)

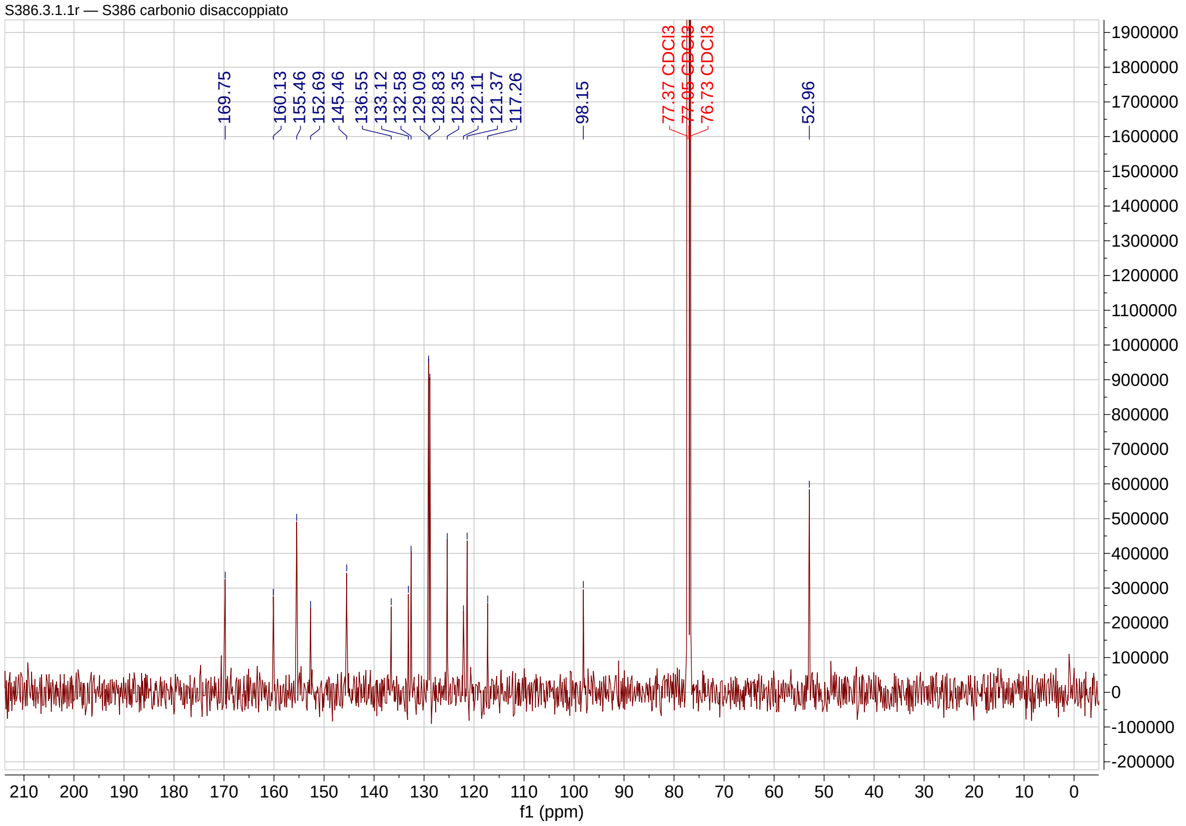

^{1}HNMR compound **8f** ($CDCl_3$)

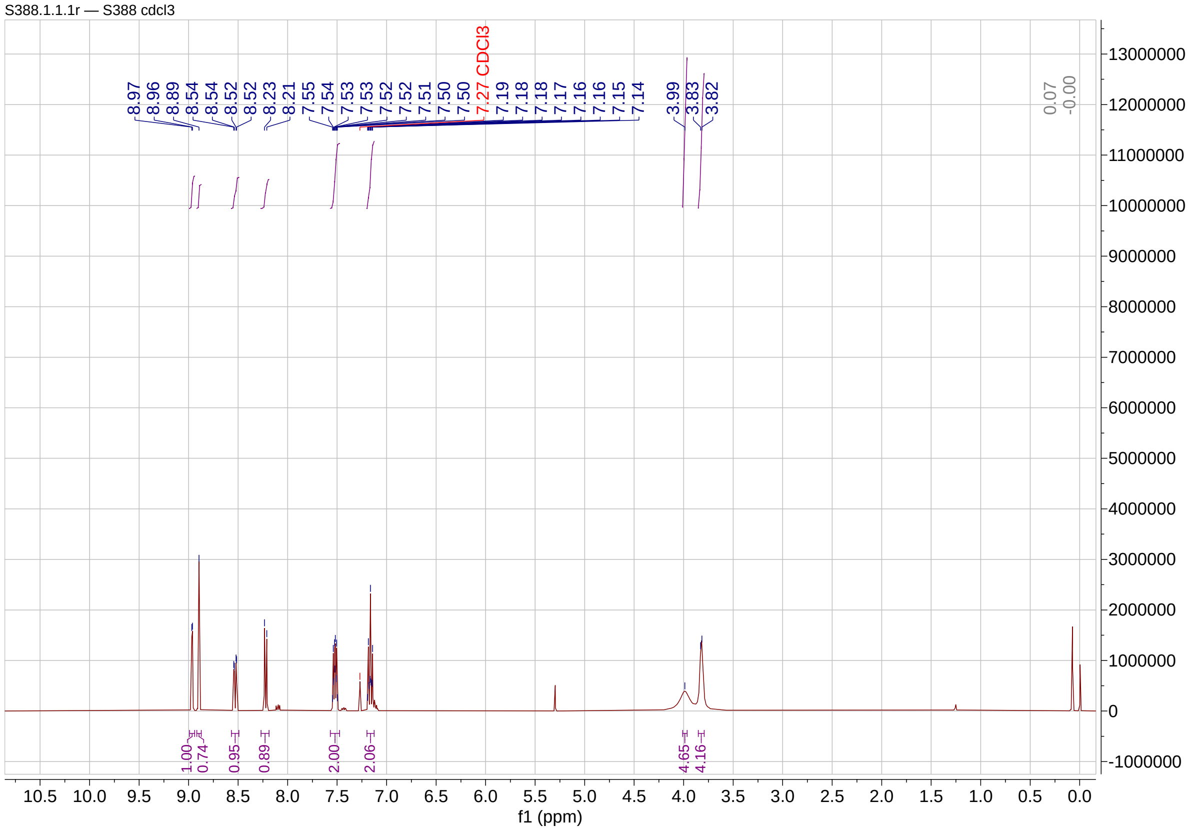

^{13}CNMR compound **8f** ($CDCl_3$)

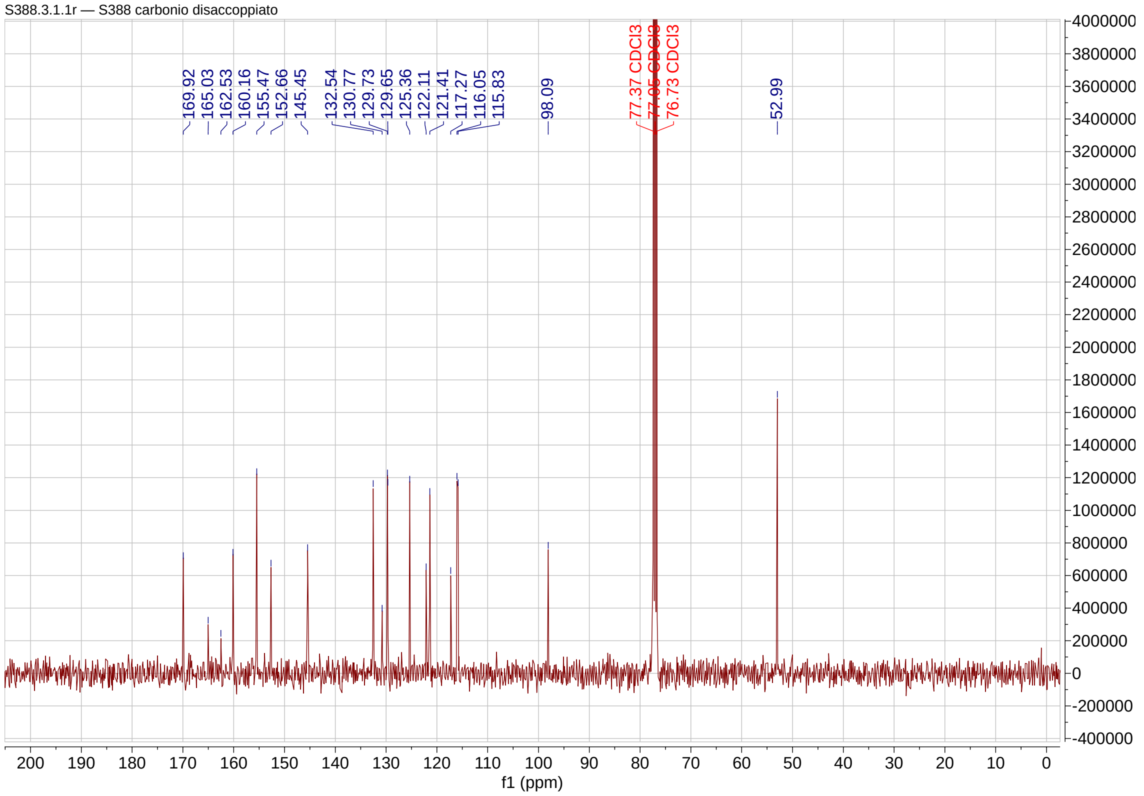

^{1}HNMR compound **8g** ($CDCl_3$)

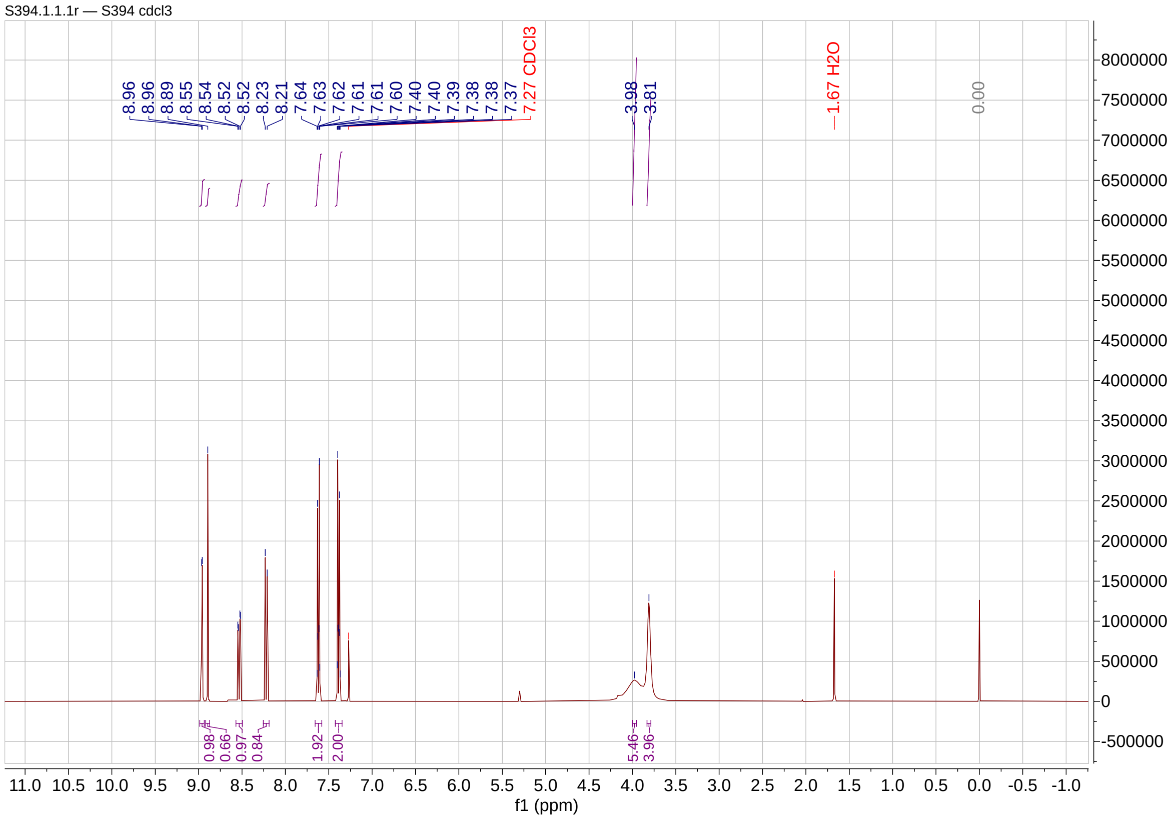

^{13}CNMR compound **8g** ($CDCl_3$)

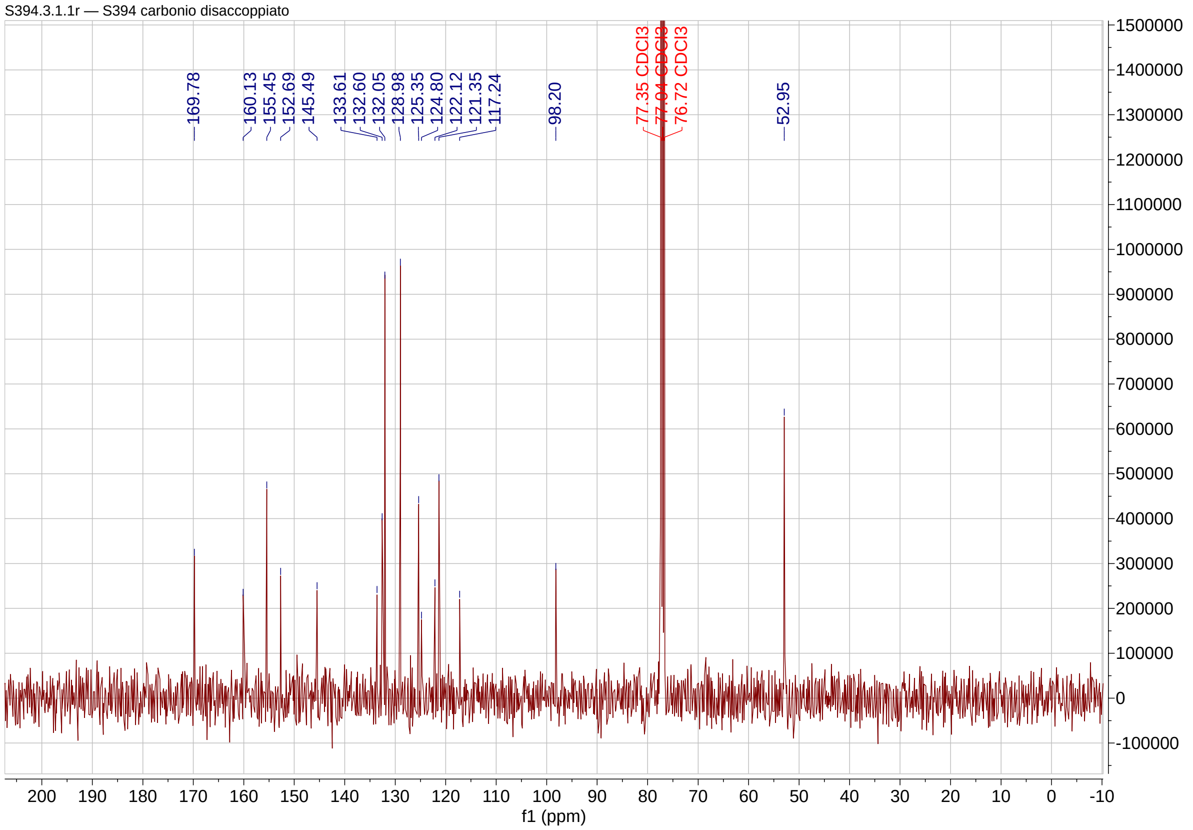

^{1}HNMR compound **8h** ($CDCl_3$)

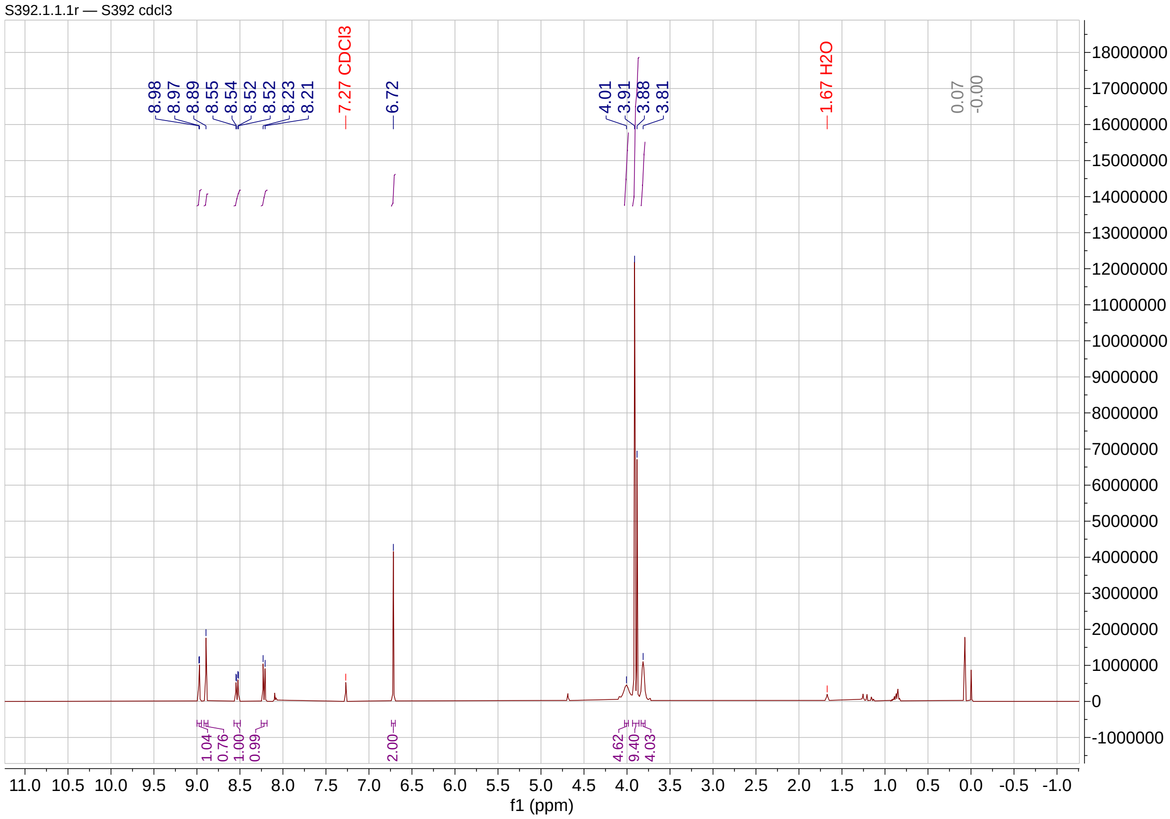

^{13}CNMR compound **8h** ($CDCl_3$)

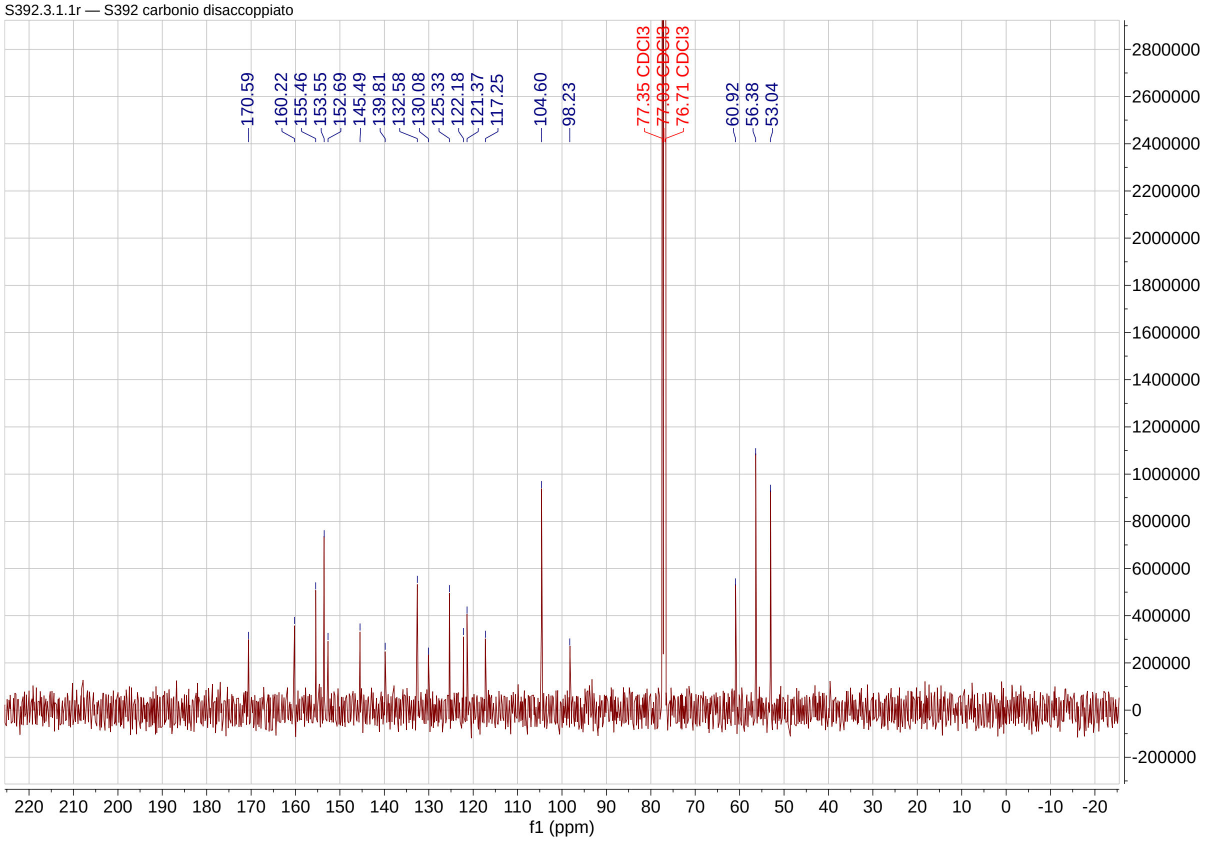

^{1}HNMR compound **8i** ($CDCl_3$)

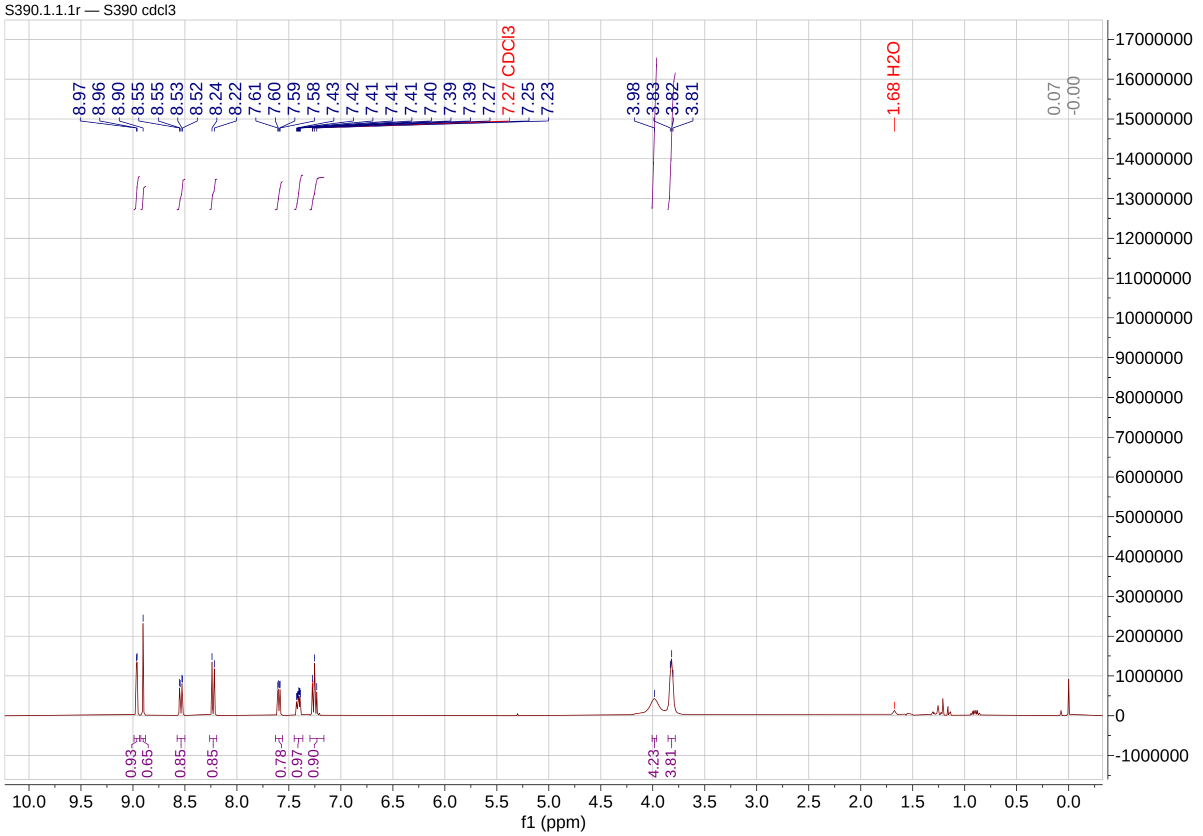

^{13}CNMR compound **8i** ($CDCl_3$)

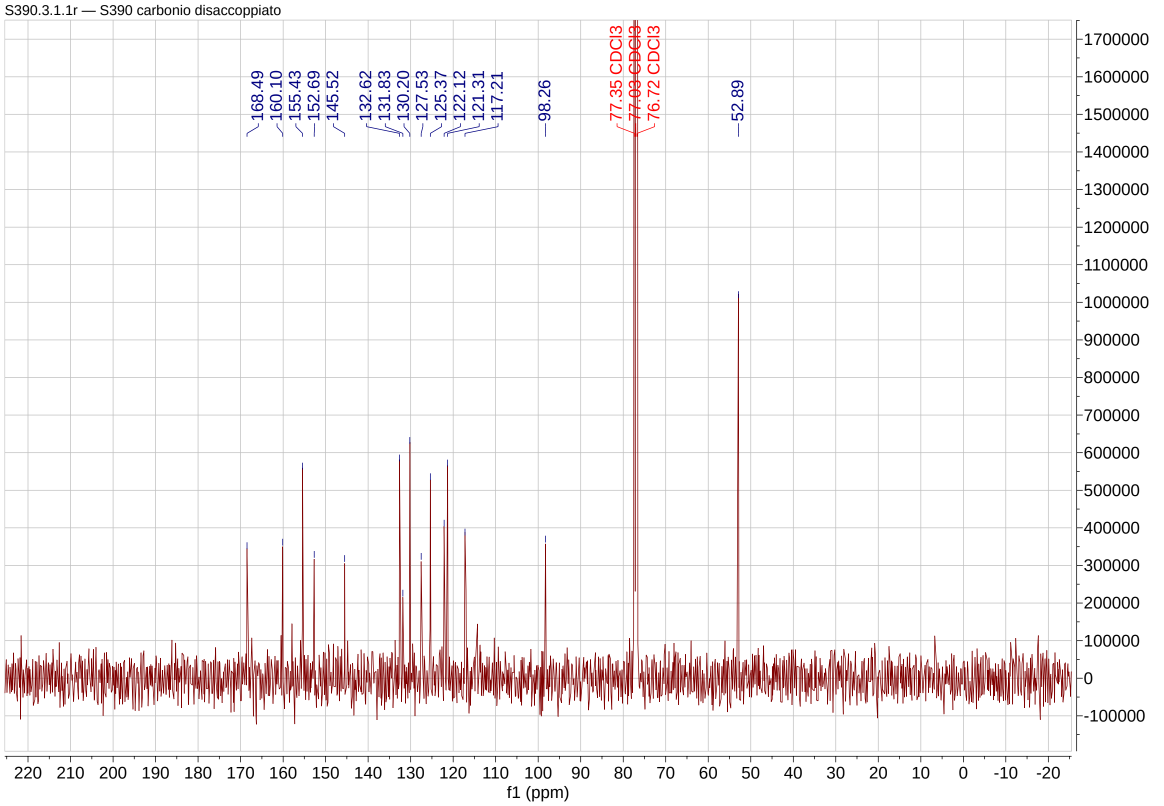

^{1}HNMR compound **8l** ($CDCl_3$)

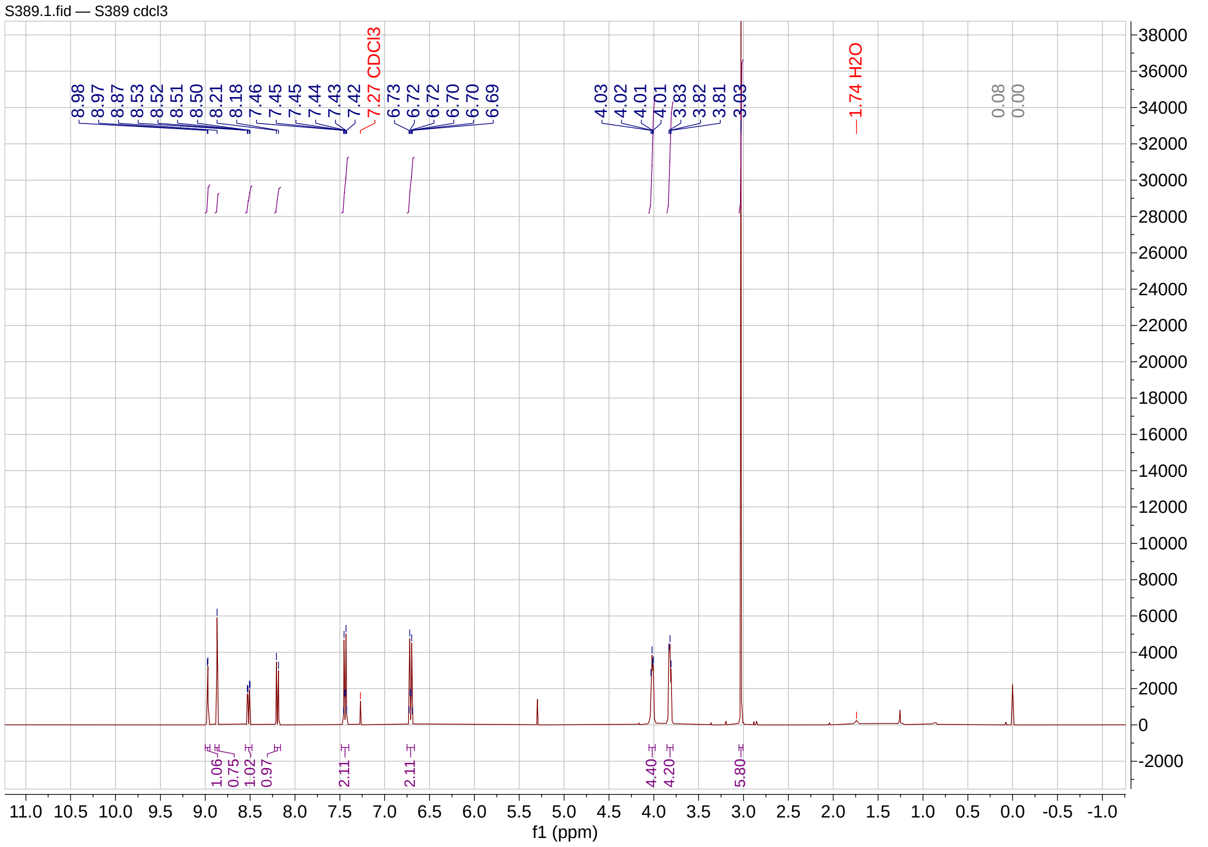

^{13}CNMR compound **8l** ($CDCl_3$)

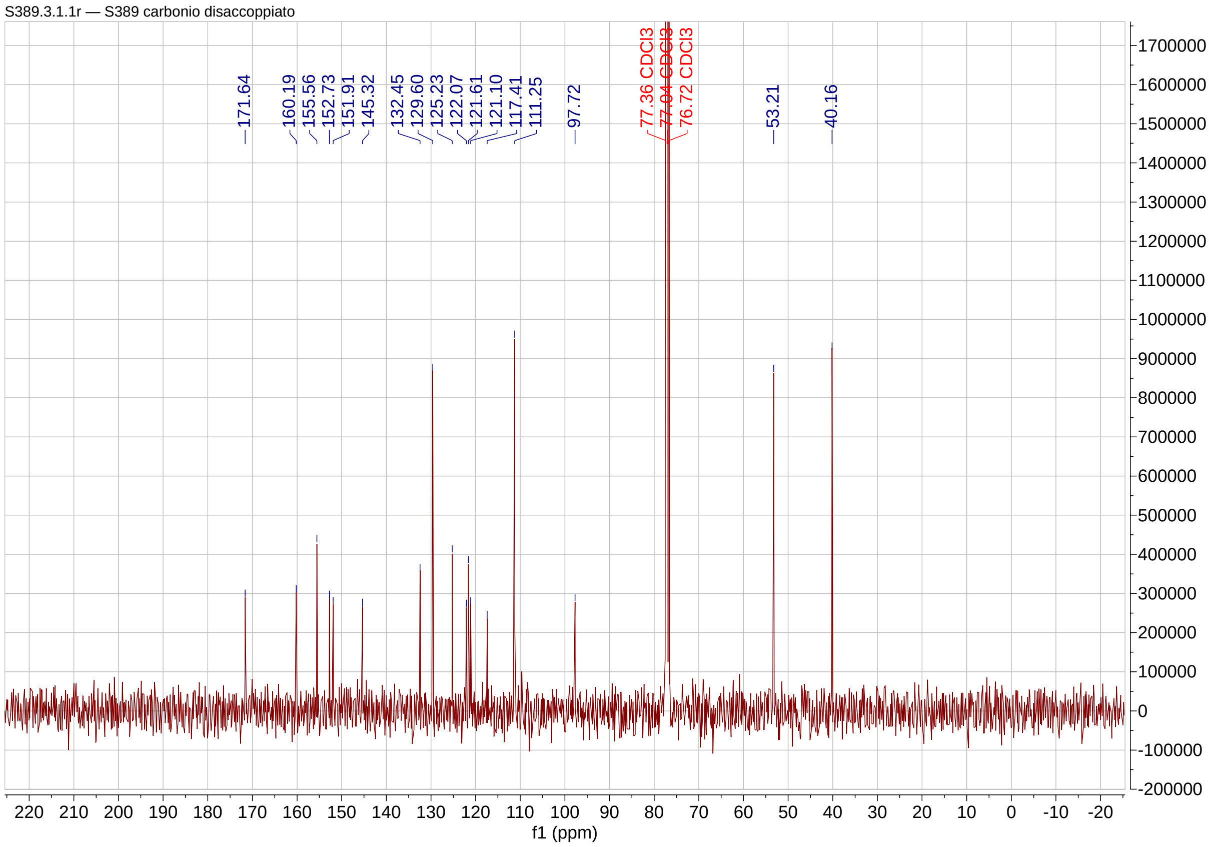

Supplement: Supplementary file 1 — Supporting Information [file OPEN-14-e202400518-s001.pdf]
